# Arsenic Removal from Groundwater by Solar Driven Inline-Electrolytic Induced Co-Precipitation and Filtration—A Long Term Field Test Conducted in West Bengal

**DOI:** 10.3390/ijerph14101167

**Published:** 2017-10-02

**Authors:** Philipp Otter, Pradyut Malakar, Bana Bihari Jana, Thomas Grischek, Florian Benz, Alexander Goldmaier, Ulrike Feistel, Joydev Jana, Susmita Lahiri, Juan Antonio Alvarez

**Affiliations:** 1 AUTARCON GmbH, D-34117 Kassel, Germany; benz@autarcon.com (F.B.); goldmaier@autarcon.com (A.G.); 2 International Centre for Ecological Engineering, University of Kalyani, Kalyani, West Bengal 741235, India; pradyutmalakar2@gmail.com (P.M.); minku_lahiri@yahoo.co.in (S.L.); 3 Kalyani Shine India, Kalyani, West Bengal 741235, India; bbjana@gmail.com; 4 Division of Water Sciences, University of Applied Sciences Dresden, D-01069 Dresden, Germany; thomas.grischek@htw-dresden.de (T.G.); ulrike.feistel@htw-dresden.de (U.F.); 5 Public Health Engineering Department, Kalyani, West Bengal 741235, India; jana_joy1969@rediffmail.com; 6 AIMEN, C/. Relva, 27 A—Torneiros, Porriño, 36410 Pontevedra, Spain; jaalvarez@aimen.es

**Keywords:** arsenic removal, electro-chlorination, oxidation, co-precipitation, chlorination, decentralized drinking water supply

## Abstract

Arsenic contamination in drinking water resources is of major concern in the Ganga delta plains of West Bengal in India and Bangladesh. Here, several laboratory and field studies on arsenic removal from drinking water resources were conducted in the past and the application of strong-oxidant-induced co-precipitation of arsenic on iron hydroxides is still considered as the most promising mechanism. This paper suggests an autonomous, solar driven arsenic removal setting and presents the findings of a long term field test conducted in West Bengal. The system applies an inline-electrolytic cell for in situ chlorine production using the natural chloride content of the water and by that substituting the external dosing of strong oxidants. Co-precipitation of As(V) occurs on freshly formed iron hydroxide, which is removed by Manganese Greensand Plus^®^ filtration. The test was conducted for ten months under changing source water conditions considering arsenic (187 ± 45 µg/L), iron (5.5 ± 0.8 mg/L), manganese (1.5 ± 0.4 mg/L), phosphate (2.4 ± 1.3 mg/L) and ammonium (1.4 ± 0.5 mg/L) concentrations. Depending on the system setting removal rates of 94% for arsenic (10 ± 4 µg/L), >99% for iron (0.03 ± 0.03 mg/L), 96% for manganese (0.06 ± 0.05 mg/L), 72% for phosphate (0.7 ± 0.3 mg/L) and 84% for ammonium (0.18 ± 0.12 mg/L) were achieved—without the addition of any chemicals/adsorbents. Loading densities of arsenic on iron hydroxides averaged to 31 µgAs/mgFe. As the test was performed under field conditions and the here proposed removal mechanisms work fully autonomously, it poses a technically feasible treatment alternative, especially for rural areas.

## 1. Introduction

Arsenic is a naturally occurring element that is present in food, soil, air and water and is known to be highly toxic. Humans are exposed to different arsenic species, whereas the dissolved inorganic forms in drinking water are most significant for natural exposure and are the main cause for adverse effects on human health. Chronic arsenic toxicity ranges from various forms of skin disease to damage of internal organs, cancer and can ultimately lead to death [1,2]. First symptoms of chronic arsenic poisoning appear after 5–15 years of drinking highly contaminated water [3]. There is no effective medical treatment for chronic arsenic poisoning [2].

### 1.1. Origin of Arsenic in Drinking Water

**Figure 1:**
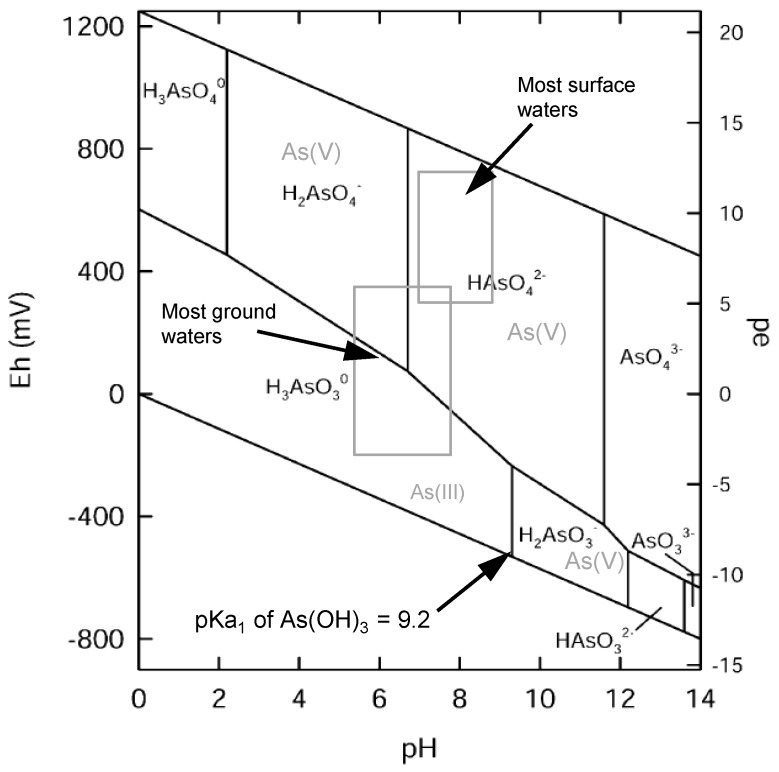
Eh-pH chart of aquatic arsenic species under oxic conditions at 1 bar and 25 °C. Reprinted and adapted from [9] with permission from Elsevier.

The primary source of arsenic in groundwater is of geogenic origin. Under oxidizing conditions in near to surface environments arsenic is mobilized by the oxidation of arsenic-bearing minerals such as arsenopyrite, from where it is released and deposited into sediments [4]. The most significant geochemical trigger releasing this arsenic again from those sediments into groundwater is the reductive dissolution of arsenic-bearing Fe(III)oxides to which the arsenic was originally adsorbed [5]. The pH value, the presence of adsorbents such as oxides and hydroxides of Fe(III), Al(III), Mn(III/IV), competing desorbents, dissolved organic matter and humic substances as well as clay minerals influence the quantities and species of arsenic that are released into the groundwater [4,6]. In oxic and circum-neutral environments mostly found in surface waters pentavalent arsenate as H_2_AsO_4_^−^ is the dominant oxidation state [4]. In suboxic conditions, as found in groundwater, arsenate (as H_2_AsO_4_^−^, and HAsO_4_^2−^) as well as trivalent arsenite in the form of arsenous acid (H_3_AsO_3_^0^) coexist below a pH of 9 (Figure 1) [4,7,8,9]. In anoxic milieu arsenic mobility is high because As(III) is less strongly sorbed onto oxides than As(V) [10] and the dissolved trivalent arsenic is reported to be 60 times as toxic as the oxidized pentavalent form [2]. Organic arsenic species are rarely present in drinking water at concentrations >1 µg/L and considered less significant compared to inorganic species in water treatment [8].

### 1.2. Total Number of People Affected

The exposure to arsenic through the consumption of groundwater is a problem, especially in South Asia [11], South-East Asia [12,13,14] and Latin America [15,16]. The mostly affected region worldwide is the Ganga-Meghna-Brahamaputra plain in Bangladesh and West Bengal, India. In Bangladesh three out of five million wells drilled into Ganga alluvial deposits and used for public water supply are assumed to be contaminated by arsenic released from the aquifer sediments. Up to 70 million people in Bangladesh [3,17] and 42 million people in West Bengal are affected [3]. The WHO guideline value for arsenic was reduced in 1993 from 50 to 10 µg/L and is considered as provisional. Whereas most countries have taken the new WHO guideline value into their legal frameworks, in Bangladesh a total arsenic concentration of <50 µg/L is still permissible. In India this guideline value is acceptable “if no other sources is available” [18,19]. The inhabitants of rural areas are mostly prone to consume contaminated water as they rely on shallow tube wells [20].

### 1.3. Arsenic Removal Plant (ARP) and Operational Challenges

**Figure 2:**
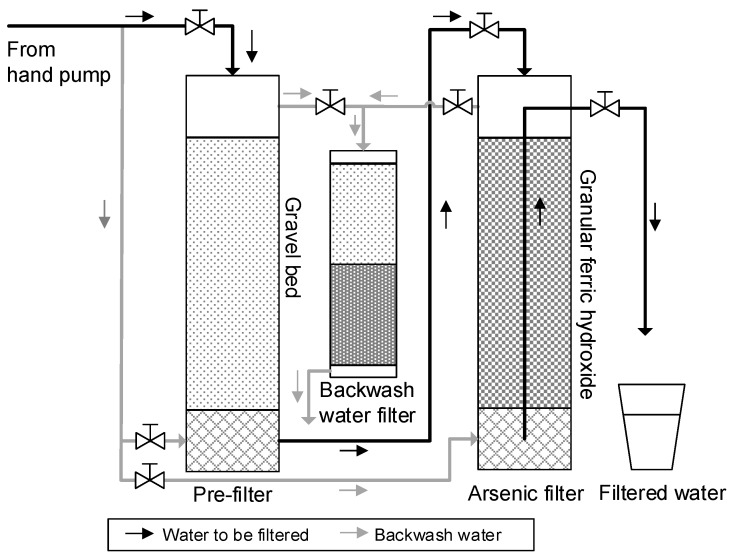
Scheme of a commonly applied ARP in West Bengal and Bangladesh (after [22]) [1].

Co-precipitation and adsorption for an efficient arsenic removal are long known and well documented in the literature for lab scale tests. Still, solutions that apply these principles in the field have failed, mainly due to the requirements for reagent dosing, the exchange of adsorption media and the non-compliance of users. In West Bengal and Bangladesh different low-tech systems for arsenic mitigation are in use (e.g., Sonofilter, Pal Trockner, Bucket Treatment Unit, Steven Institute Technology, Three Kolshi) and a typical arsenic removal plant (ARP) applied is shown in Figure 2 [21,22]. A two year systematic study conducted in South 24 Parganas District in West Bengal showed poor performance and reliability of 11 out of 18 ARPs produced by national and international suppliers [23]. A more comprehensive study conducted throughout West Bengal showed that 475 out of 570 ARPs were not useful, with 145 not even in working conditions. Removal of arsenic by 264 ARPs showed that arsenic contamination could be reduced from 185 ± 165 µg/L to 44 ± 87 µg/L (76%). 28% of these stations could not meet the guideline value of 50 µg/L and only 39 out of 213 ARPs could remove iron below 0.3 mg/L with mean values of 2.63 ± 4.59 mg/L in the filtered water [24]. Only 30% of 432 investigated units received frequent backwash with only ten stations being backwashed as given per instructions for safe arsenic removal [24]. Further, operational problems included clogging of the filter beds mainly caused by sand gushing through the use of hand pumps and short exchange intervals of adsorption media as maturity was generally reached earlier than predicted. Acceptance by users has been hampered due to increased water collection times, manual pumping, malodorous, colored water and defunct units [23,24]. In a different study from West Bengal 34 community tube wells, each equipped with one out of four types of ARPs, were analyzed by [25] and removal capacities of only 71% (PalTrockner using MnO + silica gel + gravel + sand), 42% (Anir Engineers using activated Al_2_O_3_), 42.5% (Amal Filter, using activated Al_2_O_3_) and 23% (ion exchange using ion exchange resin) were achieved. Another commonly applied removal approach requires the addition of pre-packed chemicals for oxidation and co-precipitation into plastic buckets filled with the contaminated water. The systems performed well considering arsenic removal rates, when consumers complied with given procedures. However a constant supply and addition of chemicals [26] is required and the filters are prone to enrichment of coliforms [27].

These studies show that feasible approaches to assure continuous operation and constant arsenic removal rates under the challenging conditions are still missing. When dissolved iron is available in the groundwater the pre-oxidation and coagulation-precipitation is still considered to be the most cost effective technological approach for the mentioned regions [28,29]. The following explanations focus on this process under the application of chlorine as oxidant and precipitated iron hydroxide as adsorbent because it is the underlying removal approach of the here presented study.

### 1.4. Oxidation of Arsenic

The oxidation of As(III) to As(V) not only reduces the toxicity of the arsenic, but is a pre-requisite for a successful co-precipitation with iron hydroxide [22]. Whereas with atmospheric oxygen Fe(II) can be oxidized within minutes to Fe(III), As(III) oxidation is very slow, requiring days or weeks [10]. Pure oxygen reduces the oxidation time to a magnitude of hours or days, whereas ozone, permanganate, Fenton’s reagent, and hypochlorous acid can achieve complete oxidation within a matter of minutes [22,30]. Chlorine dioxide has shown only limited capabilities of oxidizing As(III) and monochloramine was ineffective [31,32]. An overview on the advantages and disadvantages of conventionally available arsenic oxidants is found in [33] and summarized in Table 1.

Reference [32] identified extremely fast reaction half times t_1/2_ of 95 ms and 11 ms for the oxidation of As(III) by chlorine and ozone respectively at a pH of 7 and an oxidant concentration of 2 mg/L:

(1) $$H_{3}AsO_{3}+HClO\to H_{2}{AsO_{4}}^{-}+Cl^{-}+H^{+}$$

For chlorine the reaction rate hereby depends on the varying contributions of the As(III) and the chlorine species available at a given pH. The dissociation constants for the relevant reactions are as follows:

(2) $$As{\left(OH\right)}_{3}\overset{K_{a1}}{↔}As{\left(OH\right)}_{2}O^{-}+H^{+}\;pK_{a1}=9.2$$

(3) $$HOCl\;\overset{K_{a}}{↔}OCl^{-}+H^{+}\;pK_{a}=7.4$$

**Figure 3:**
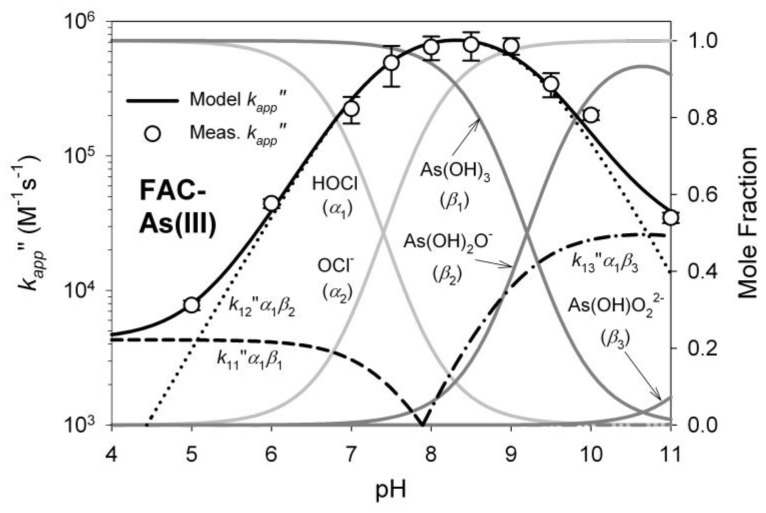
Apparent second-order rate constant for As(III) oxidation by chlorine. Reprinted from [32] with permission from American Chemical Society.

As(OH)_2_O^−^ is expected to be a stronger nucleophile than its counterpart As(OH)_3_, whereas HOCl is the by far stronger oxidizing agent than OCl^−^ [32,34]. The reaction rate therefore is highest near pH = 8.3 (being the average of both pK values) where the product of the molar fraction ratio of the reagents α_HOCL_ and β_As(OH)2O_^−^ reaches its maximum (Figure 3).

At lower pH, higher redox potentials are required for As(III) oxidation (Figure 1). When chlorine is used as oxidizing agent this effect is compensated as with decreasing pH HOCl becomes predominant. Therefore, chlorine can assure high oxidation rates of arsenite for the pH ranges relevant in drinking water supply.

### 1.5. Co-Precipitation with Iron Hydroxide and Ions Competing with Adsoption Sites

The co-precipitation of arsenic on iron hydroxides is an effective method for removing arsenic from water [34,35,36]. Freshly formed iron hydroxide particles show a sufficiently high adsorption capacity than particles already a few minutes old [22,35,37], because ferrihydrite particles formed during oxidation have more surfaces sites. Here the removal is controlled by co-precipitation involving adsorption as well as entrapment of arsenic by inclusion in growing particles, whereas crystallites of pre-formed particles allow only surface adsorption [38]. The maximum adsorption densities of iron hydroxide that were formed during the presence of arsenic compared to pre-formed iron hydroxides were analyzed by [36,38] and found to be three and five times higher, respectively. Studies have further shown that particles formed during the oxidation with chlorine had a by 40% higher capacity to remove As(V), than particles formed from oxidation with oxygen alone [29]. Further, iron hydroxides particles have a higher adsorption capacity for As(V) than As(III) [10]. The simultaneous oxidation of Fe(II) (Equation (4)) and As(III) (Equation (1)) using strong oxidants therefore has the capacity to achieve high removal rates:

(4) $$2Fe^{2+}+HOCl+3H_{2}O↔2FeO\left(OH\right)↓+Cl^{-}+5H^{+}$$

**Figure 4:**
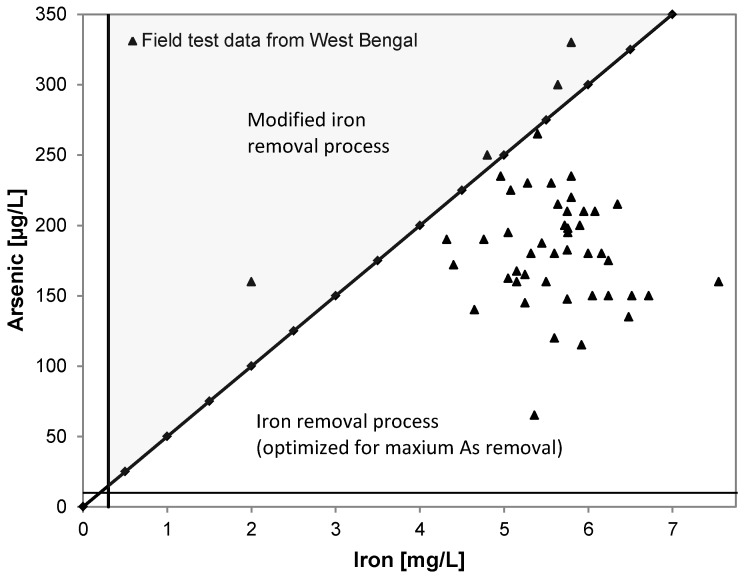
Selection guide for arsenic removal strategy in dependence on initial arsenic and iron concentration in water and Fe/As ratios found during the here conducted field test (after [29]).

The Fe/As ratio has a major influence on the removal efficiency by co-precipitation. In lab experiments mass ratios above 20:1 [29] (Figure 4) and 25:1 [37] and the addition of chlorine have been required to reduce arsenic concentrations to below 10 µg/L.

Phosphate and silicate have been identified as the major anions affecting the removal of arsenic by co-precipitation with ferric chloride as they compete primarily for a similar set of surface sites [13,22]. Reference [26] assumes a surface site loading density of 0.9 mol sites/molFe for competing ions.

**Figure 5:**
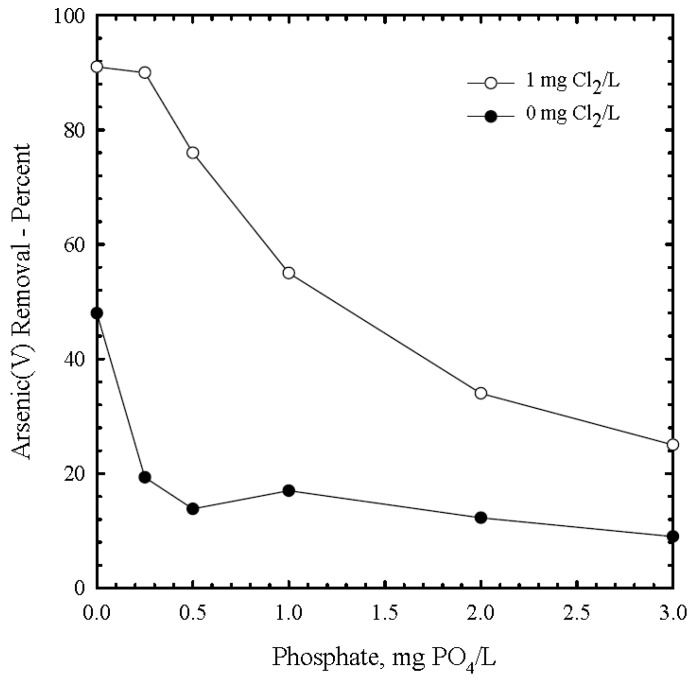
Effect of chlorine and phosphate on arsenic(V) removal with iron(III) (10 mg DIC/L, 1 mg Fe(II)_initial,_ pH = 8, As(V) 100 µg/L [28].

In a study conducted in Bangladesh with elevated phosphate (1.6–2.7 mg/L) and silicate concentrations (14–20 mg/L) in groundwater a Fe/As mass ratio as high as 40:1 (20 mg/L Fe, 500 µg/L As) was required to achieve concentrations <50 µg/L and arsenic removal rates of 85% resulting in an iron-hydroxide loading rate of 21 µgAs/mgFe. The same study showed that natural groundwater containing nearly no phosphate and little silicate (PO_4_-P: 0.02 mg/L, Si: 6.6 mg/L) a Fe/As mass ratio of only 12:1 (4.8 mg/L Fe, 400 µg/L As) was sufficient to achieve nearly 100% removal of arsenic, resulting in a loading rate of 83 µgAs/mgFe. Spiking the same water with phosphate and silicate to achieve the abovementioned competing ion concentrations (PO_4_-P: 1.9 mg/L, Si: 18.8 mg/L) arsenic removal rates of only 40% were achieved and sludge loading densities reduced to 33 µgAs/mgFe. In similar studies the adsorption density of the iron was reduced from 95 µg/mg Fe(II) to 32 µg/mg Fe(II) after increasing phosphate from 0 to 3 mg/L (at 100 µg/As(III), 1 mg Fe(II) initial, pH = 8, Cl_2_ = 1 mg/L). When no chlorine was used arsenic removal rates of only 50% and loading densities of 50 µgAs/mgFe were reached but dropped to 20% and 12 µgAs/mgFe after adding only 0.5 mg/L of phosphate (Figure 5) [29].

A study conducted in Bangladesh with bucket household treatment units required Fe/As mass ratios between 35:1 and 164:1, through the addition of 1.5 g of packed ferric sulfate ((Fe_2_(SO_4_)_3_) and 0.5 g of calcium hypochlorite (Ca(OCl)_2_) per 20 L bucket. Half of the samples (three out of six) met the WHO guideline value [34].

At natural pH a Fe/P mass ratio of 2.7:1–3.6:1 (molar Fe/P ratio 1.5:1–2.0:1) is required to remove phosphate, and only iron in excess of this ratio is available to remove arsenic [13]. The same study suggested a Fe/As mass ratio of 40:1 through the addition of FeCl and hypochlorite for complete As(III) oxidation to achieve arsenic concentrations below 50 µg/L considering waters of unfavorable phosphate concentrations as found in Bangladesh. Those waters are similar to the ones found during this study in West Bengal. While carbonate also exerts a negative effect by competing with arsenic sorption, calcium has a positive effect by increasing phosphate sorption and precipitation together with iron. As the concentrations of silicate, calcium and carbonate do not differ as much in different regions, and their effects are more difficult to quantify, the Fe/As and Fe/As ratios are the most important factors for evaluating arsenic removal [13].

### 1.6. pH Dependency

Co-precipitation is strongly pH dependent, with generally higher adsorption densities at lower pH values [29,36]. As(V) removal during iron co-precipitation has shown to be less affected by pH ≤ 8, when chlorine is used as oxidant. A study conducted by [39] showed that arsenic removal decreased from nearly 95% to below 30% at a respective pH increase from 8 to 10. As(V) removal as Fe(III) co-precipitate decreased from 95% to 0% when pH was increased from 6 to 10 (As(V) = 100 µg/L, Fe(III) = 1.5 mg/L) [26]. However, pH values above 8 are not of interest in groundwater treatment and when chlorine is applied due to the increasing concentration of OCl^−^ (Figure 2). Compared to alum-based coagulants iron-based coagulants are more efficient under a wider variety of pH conditions and especially advantageous at pH values above 7.6 [21,22].

### 1.7. Self-Sufficient Treatment Approach for Autonomous Arsenic Removal

The here presented approach for arsenic removal is based on the above described mechanism for an efficient co-precipitation and is integrated into a more advanced technical but very robust setting. This includes electrical pumping under anoxic conditions, inline-electrolytic production and in situ application of strong oxidizing agent for oxidation of Fe(II) to Fe(III) and As(III) to As(V), co-precipitation, subsequent filtration with automatic filter media backwash and water quality monitoring by means of ORP reading. This combination has the potential to achieve satisfying arsenic removal rates without the usual addition of chemicals [40]. It further can solve several of the above described operational challenges such as oxidant supply, dosing, exchange of adsorbents and user compliance. The motivation of this work is to test the setting for the first time in a real case scenario in order to prove high arsenic removal rates and long term reliability. The complete setting can be run on solar PV panels, allowing its application independent of any technical infrastructure. The pilot system was installed and operated in Nadia District, one of the most severely arsenic effected regions of West Bengal [25], allowing the evaluation of its arsenic removal efficiencies and operability in a real case scenario under challenging groundwater conditions.

## 2. Materials and Methods

### 2.1. Inline Electrolysis

The SuMeWa|SYSTEM^®^ (from sun meets water) produced by AUTARCON GmbH (Kassel , Germany) was originally designed for the disinfection of drinking water in rural developing regions through the in situ production of chlorine [41]. This process is mediated through inline-electrolysis of the natural chloride content of the water using dimension stable titanium electrodes coated with iridium and ruthenium oxides (MOX-electrodes) and does not require any addition of chemicals.

Chlorine gas is produced at the electrolytic cell following the given reactions:

Anodic reaction chlorine:

(5) $$2Cl^{-}↔Cl_{2}+2e^{-}$$

Anodic reaction oxygen:

(6) $$2H_{2}O↔O_{2}+4H^{+}+4e^{-}$$

Cathodic reaction:

(7) $$2H_{3}O^{+}+2e^{-}↔H_{2}+2H_{2}O$$

The chlorine gas rapidly dissociates in water to hypochlorous acid, being chemically the same oxidizing agent as in conventional chlorination systems [42]:

(8) $$Cl_{2}+2H_{2}O↔HClO+Cl^{-}+H_{3}O^{+}$$

The chlorine production capacity depends on a multitude of factors, among them chloride concentration, current density, temperature and chlorine demand of the water [43] and has to be considered when operating such a reactor. The SuMeWa|SYSTEM^®^ is especially suited for small scale applications in areas where no constant supply and dosing of chlorine can be assured.

**Figure 6:**
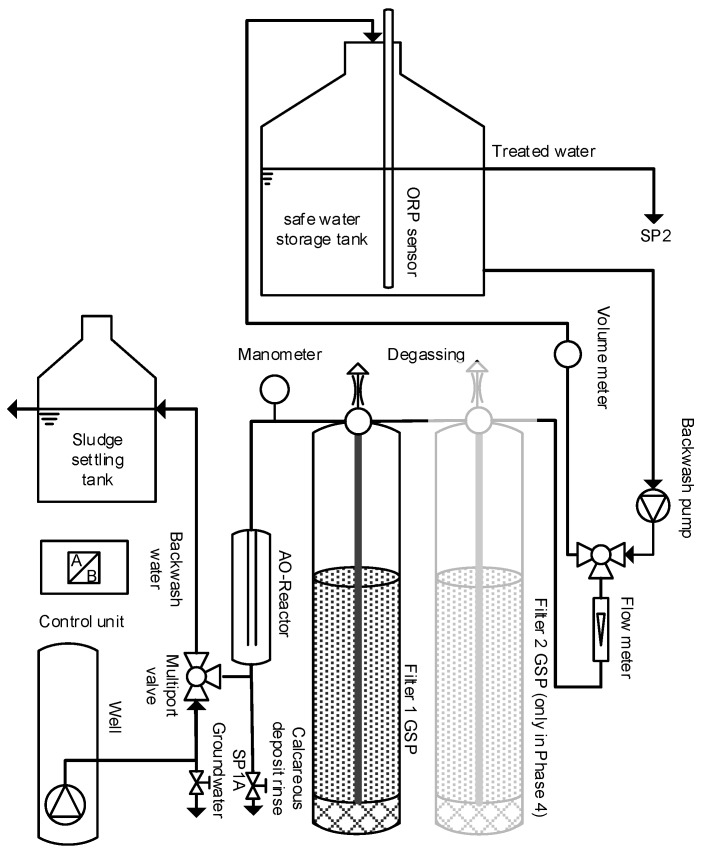
Pilot setting in West Bengal.

For this pilot test the system was extended with a pressurized vessel containing filtration media for arsenic removal. Contaminated water was pumped through the electrolytic reactor and the filter into a final water storage tank (Figure 6). A flow sensor (GEMÜ 850, GEMÜ Gebr. Müller, Ingelfingen-Criesbach, Germany) and integrated control unit assured constant flow through the system during pressure build up along the filtration process.

The electrolytic cell stack has a total surface area of 600 cm² and was operated under galvanostatic conditions with a maximum current of 5 A resulting in a current density of 17 mA/cm². At a flow rate of about 60 L/h the maximum specific load accounted to 80 mAh/L. For the removal of calcium deposits at the cathode the polarity was inversed in intervals of 60 min. To prevent insufficient chlorine production and subsequent failure of Fe(II), As(III) and Mn(II) oxidation breakpoint chlorination should be applied. Therefore the stoichiometric relationships between chlorine and the reductants in Table 2 had to be considered.

The system was operated in four phases (P1, P2, P3, P4) under different current densities of 14 mA/cm² (P1), 16.5 mA/cm² (P2 and P4) and 12 mA/cm² (P3). Sufficient oxidant supply was expected to be assured whenever free available chlorine (FAC) was present and ORP reached values ≥500 mV in the final storage tank. Between P3 and P4 the system was paused and extended with a second pressure vessel.

### 2.2. Filtration

The in situ formed iron precipitates with the adsorbed As(V) were retained in one (P1, P2, P3) pressurized filter with a bed depth of 66 cm and a diameter of 17.7 cm accounting to a media volume of 16.3 L (23 kg). For P4 an identical second filter was placed in series as a backup for potential filter breakthrough. As filter media GSP^®^ (Inversand, Clayton, NJ, USA), a manganese dioxide coated sand was used. The MnO_2_ coating was expected to act as an intermediate redox buffer in case of sporadically insufficient chlorine production e.g., during polarity inversion of the electrolysis cells, as the coating is capable in mediating the oxidation of iron, manganese, and arsenic as well [31]. Reduced manganese oxides are regenerated by excessive chlorine production keeping the media constantly in a regenerated state (Equation (9)):

(9) $$HOCl+H_{2}O+Mn^{2+}⇌MnO_{2}\left(s\right)+Cl^{-}+3H^{+}$$

The media was supported by a 15 cm gravel layer. Freeboard for bed expansion during backwash was set to 32 cm (Table 3).

In order to prevent filter breakthrough and the desorption of removed As(V) due to progressing crystallite growth, the filters were backwashed daily with treated water at 30 m/h. In order to reduce discharge of arsenic into the environment as much as possible, backwash sludge was collected in an intermediate storage tank for settling. Surplus water was discharged after controlling its water quality.

### 2.3. Study Site

The station was installed in a densely populated village (Chandamari) close to the Kalyani University Campus in Nadia District in West Bengal. The site is located in the floodplains of the Hooghly River, a distributary of the Ganga River about 40 km from Bangladesh. A well with a depth of 12 m and a diameter of 15 cm was drilled. The filter screen is located between 6 and 12 m below ground level. By the time of installation the water table was about 3 m below ground level (bgl) and the pump was placed at 8 m bgl. Groundwater quality data are presented in Table 4 together with reference literature data from Bangladesh.

The groundwater showed high arsenic and iron concentrations with a mean Fe/As mass ratio of 32:1. Phosphate and ammonium concentrations were high but typical for these regions.

### 2.4. Sampling, Water Analysis and Monitoring 

Water samples were taken at two sampling points (SP) to measure the quality of the groundwater (SP1A) and the treated water (SP2). Samples were taken twice per week during P1 and P2 and once per week during P3 and P4. Analysis was done simultaneously for both sampling points on site.

Electric conductivity (CDC 101, Hach, Düsseldorf, Germany), dissolved oxygen (LDO 101, Hach) and pH (PHC 101, Hach) were measured with a Hach HQ40d multimeter water analyser for sampling points SP1A and SP2. Oxidation reduction potential (ORP) for SP1A was also measured with HQ40d using a Hach MTC 101 probe. The ORP SP2 was measured directly with the pilot system using a tecLine Rd electrode (Jumo, Fulda, Germany). For parameters documented in Table 5 an AL410 handheld photometer (Aqualytic, Dortmund, Germany) was used.

Total arsenic was measured with an Arsenator^®^ testkit (Palintest, Gateshead, UK). For preparation of dilutions DD Milli-Q water was used. Turbidity was measured with a TN-100 turbidity meter (Eutech, Singapore). Operational parameters such as electrolytic cell current, flow rate, power consumption of the pump and filtration intervals were monitored using an online monitoring system (AUTARCON, Kassel, Germany) set up for this pilot system.

## 3. Results and Discussion

The here evaluated test phase comprised a period of ten months from September 2016 to July 2017. During this test period a total volume of 393 m³ groundwater was treated in continuous flow. This volume accounted to a daily production of 1.35 m³ treated water. The assessment of the removal capacity of the test period is discussed below.

### 3.1. Oxidant Production and Breakpoint Chlorination

**Figure 7:**
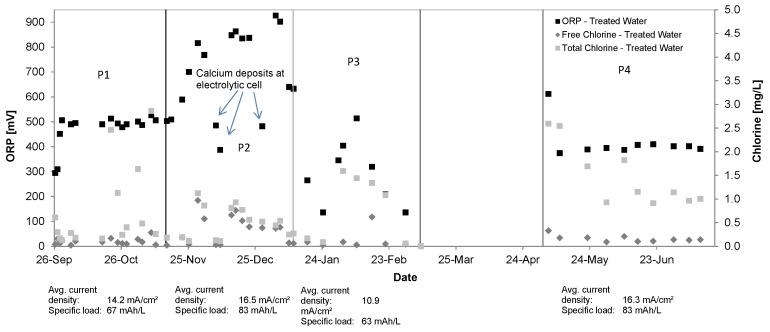
ORP, total and free chlorine concentrations in treated water, Kalyani pilot system, September 2016–July 2017.

Figure 7 shows the ORP levels (P1 = 471 ± 66 mV, P2 = 701 ± 167 mV, P3 = 291 ± 123 mV, P4 = 399 ± 176 mV) as well as the total chlorine (P1 = 0.72 ± 0.83 mg/L, P2 = 0.51 ± 0.32 mg/L, P3 = 0.82 ± 0.64 mg/L, P4 = 1.47 ± 0.62 mg/L) and free available chlorine concentrations (P1 = 0.10 ± 0.07 mg/L, P2 = 0.37 ± 0.29 mg/L, P3 = 0.14 ± 0.20 mg/L; P4 = 0.16 ± 0.07 mg/L) in the treated water (SP2) during the four operational phases.

**Figure 8:**
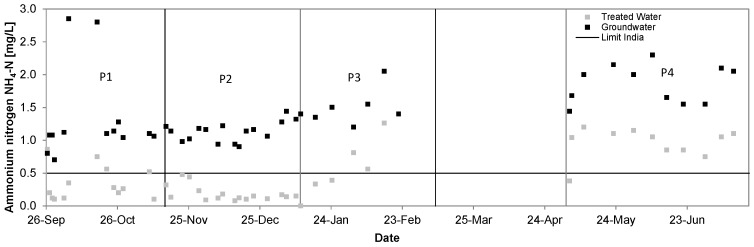
Ammonium nitrogen concentrations in groundwater and treated water during test phase, Kalyani pilot system, September 2016–July 2017.

Only during Phase 2 sufficient chlorine to achieve the chlorination breakpoint was produced, as the OPR increases substantially and FAC becomes available. Larger differences between total and free available chlorine values indicate insufficient oxidant supply, which is also represented by lower ORP values and reduced ammonium removal (see Figure 8). In all other phases the pilot system was not able to reach the chlorination breakpoint due to elevated ammonium levels.

### 3.2. Ammonium

Ammonium nitrogen levels in groundwater were reduced in P1 from 1.31 ± 0.63 mg/L to 0.34 ± 0.24 mg/L, in P2 from 1.14 ± 0.16 mg/L to 0.18 ± 0.12 mg/L, in P3 from 1.51 ± 0.27 mg/L to 0.84 ± 0.34 mg/L and in P4 from 1.86 ± 0.28 mg/L to 0.96 ± 0.23 mg/L, which accounted to a removal rate of 74%, 84%, 26% and 49%, respectively. Safe levels could only be reached, when FAC was available, as it was the case during P2. During P4 the quantity of chlorine required to reach the breakpoint was exceeded beyond the capacity of the electrolytic cell despite the same current densities of P2 and P4. This was mainly caused by the increased ammonium content, also explaining the low ORP values during P4.

### 3.3. Iron

**Figure 9:**
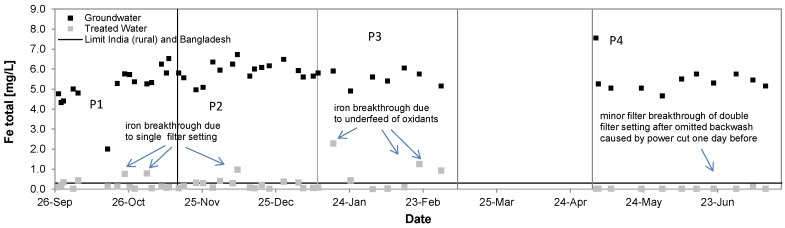
Iron removal performance of the Kalyani pilot system, September 2016–July 2017.

Figure 9 shows the removal of total iron from groundwater in P1 from 5.2 ± 1.0 mg/L to 0.2 ± 0.2 mg/L, in P2 from 5.9 ± 0.5 mg/L to 0.2 ± 0.2 mg/L, in P3 from 5.5 ± 0.4 mg/L to 0.7 ± 0.8 mg/L and in P4 from 5.5 ± 0.7 mg/L to 0.03 ± 0.03 mg/L. Removal rates accounted to 96%, 96%, 87% and >99% respectively. For Phases 1 and 2 the iron removal performance complies most of the time with the WHO and local guidelines and worked reliably.

**Figure 10:**
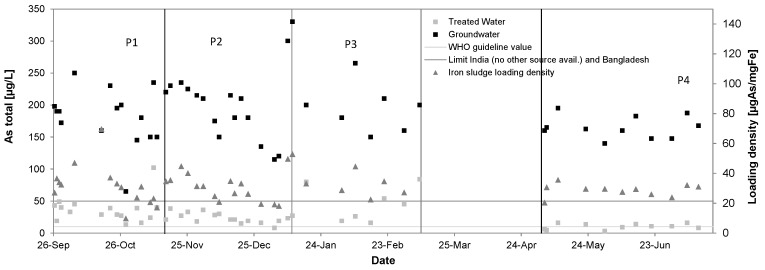
Arsenic removal performance of the Kalyani pilot system, September 2016–July 2017.

Iron removal decreased by the underfed oxidant supply during Phase 3. Filter breakthrough occurred during Phases 1–3, which also caused an increased release of arsenic (Figure 10). For the dual filter setting in Phase 4 no breakthroughs occurred and guideline values could always be maintained for iron. Under the given source water conditions the filter capacity of the dual filter setting has shown to be sufficient for the set backwash intervals.

### 3.4. Arsenic

Figure 10 shows the removal of total arsenic from groundwater in P1 from 183 ± 43 µg/L to 36 ± 20 µg/L, in P2 from 202 ± 57 µg/L to 24 ± 8 µg/L, in P3 from 195 ± 35 µg/L to 46 ± 26 µg/L and in P4 from 165 ± 17 µg/L to 10 ± 4 µg/L. Removal rates accounted to 80%, 88%, 76% and 94%, respectively. In the single filter operation only Phase 2 showed sufficiently good removal of the arsenic meeting local guidelines indicating that sufficient chlorine production improves removal performance. Fluctuations of effluent concentrations were larger when oxidant supply was insufficient. Phase 4 could show that elevated arsenic values in the treated water during P2 were caused by insufficient iron removal and filter breakthrough, as this was prevented during double filter operation.

The by weight Fe/As ratios in the source water were 31:1, 32:1, 30:1 and 34:1 and the sludge loading densities of arsenic averaged to 32 ± 13 µgAs/mgFe, 32 ± 10 µgAs/mgFe, 32 ± 7 µgAs/mgFe, 29 ± 4 µgAs/mgFe during P1–P4. Lower loading densities during P4 are related to an increased average Fe/As ratio. Figure 10 also shows that the fluctuations for the loading densities could be reduced substantially with gaining more in-field experience. The in P4 achieved effluent concentrations of arsenic (10 ± 4 µg/L) are certainly satisfying considering the given Fe/As ratios and comparing those to the by [13,27] required ratios of >40:1 to achieve concentrations <50 μg/L under real field conditions.

### 3.5. Manganese

**Figure 11:**
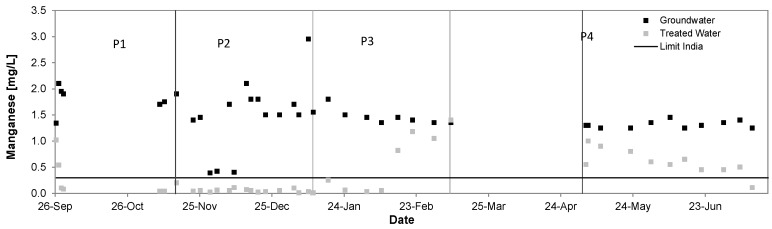
Manganese removal performance of the Kalyani pilot system September 2016–July 2017.

Figure 11 shows the removal of manganese from groundwater in P1 from 1.79 ± 0.24 mg/L to 0.30 ± 0.37 mg/L, in P2 from 1.50 ± 0.64 mg/L to 0.06 ± 0.05 mg/L, in P3 from 1.46 ± 0.14 mg/L to 0.61 ± 0.53 mg/L and in P4 from 1.31 ± 0.06 mg/L to 0.60 ± 0.23 mg/L. Removal rates accounted to 83%, 96%, 58% and 55% respectively.

**Figure 12:**
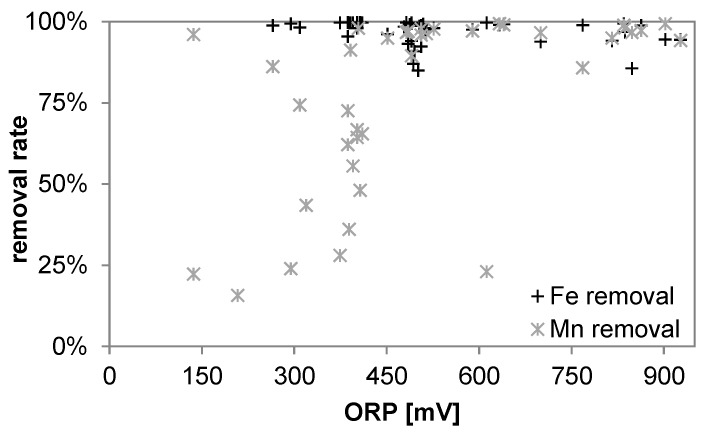
Removal rates of iron and manganese in relation ORP (values of P3 are omitted).

When for a satisfying iron removal an OPR of 250 mV was sufficient a satisfying manganese removal required ORP values >400 mV (Figure 12) This was the case only during P2 when the chlorination breakpoint was reached.

### 3.6. Phosphate

**Figure 13:**
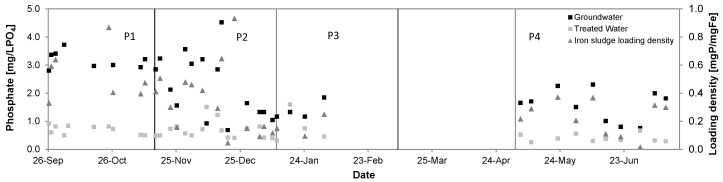
Phosphate removal performance of the Kalyani pilot system, September 2016–July 2017.

Figure 13 shows the removal of orthophosphate from groundwater in P1 = 3.5 ± 0.9 mg/L to 0.7 ± 0.2 mg/L, in P2 from 2.4 ± 1.5 mg/L to 0.7 ± 0.3 mg/L, in P3 from 1.4 ± 0.3 mg/L to 0.9 ± 0.5 mg/L and in P4 from 1.6 ± 0.5 mg/L to 0.4 ± 0.1 mg/L. Input concentrations were largely fluctuating over time. Removal rates accounted to 81%, 72%, 36% and 75%, respectively.

**Figure 14:**
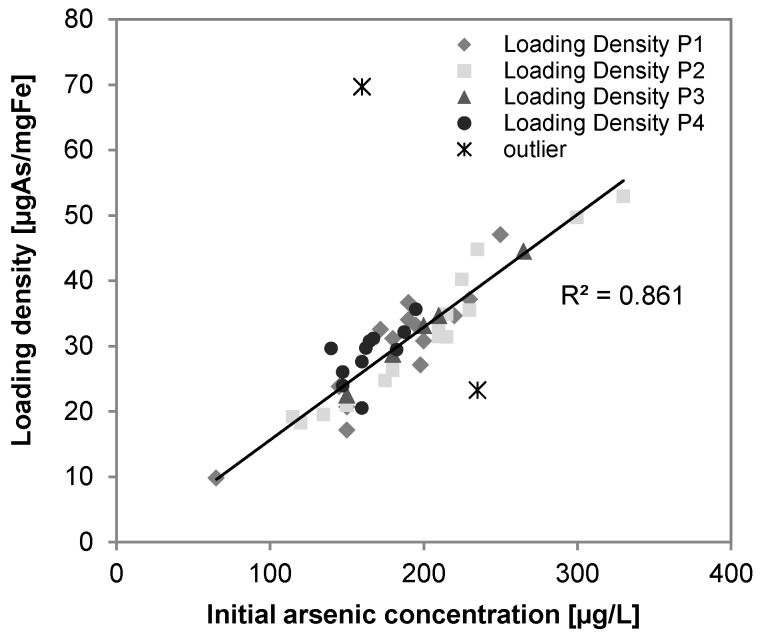
Correlation of arsenic loading densities on iron precipitates and initial arsenic concentration in groundwater (R^2^ = 0.86, *n* = 49).

**Figure 15:**
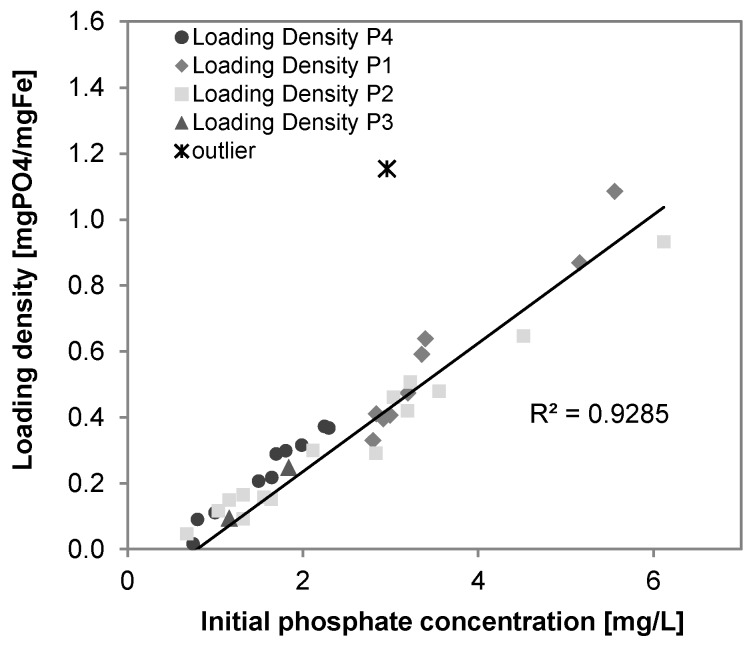
Correlation of phosphate loading densities on iron precipitates and initial phosphate concentration in groundwater (R^2^ = 0.86, *n* = 38).

Considering a surface site loading density of 0.9 mol sites/molFe [27] an average P removal of 0.6 mgP/L (19 µM) and an average iron removal 5.3 mg/L (95 µM), the loading densities averaged to 0.11 mgP/mgFe and the phosphate occupied about 20% of the adsorptive sites of the iron sludge, which is in agreement with the results stated in [26]. Figure 14 and Figure 15 show the positive correlation between the loading density of arsenic (R^2^ = 0.86, *n* = 49) and phosphate (R^2^ = 0.86, *n* = 38) on iron hydroxide.

**Figure 16:**
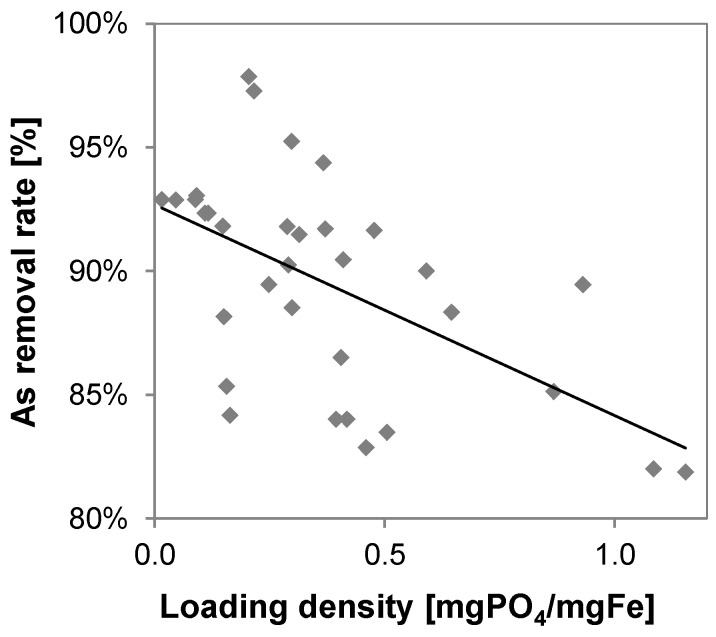
Arsenic removal in relation to phosphate loading density

Due to several overlaying effects (filter breakthrough, calcareous deposits at the electrolytic cell, fluctuations of FAC, calcium and carbonate concentration etc.) caused by the operation of the system under real field conditions arsenic and phosphate loading densities were fluctuating and no clear correlation between arsenic removal rates and phosphate loading densities on iron sludge could be identified. However, the data indicate that lower sludge loading densities of phosphate favor arsenic removal rates above 90% (Figure 16).

A summary of the water quality parameters analyzed during P1–P4 is given in Table A1.

### 3.7. Energy Demand

In Table 6 the power and energy demands for the system operation are given.

Considering a daily treatment capacity of 1.35 m³ the energy demand for the treatment process including pumping and system control accounts to 1.21 kWh/m³. For comparison the data of a study from Bihar where a small scale reverse osmosis (RO) system with a similar treatment capacity was tested, showed an electricity demand between 3 and 4 kWh/m³ only for the RO [45]. Due to different well depths the energy demand for pumping could not be directly compared. However, at the applied RO permeate recovery rate of only 10% the quantity of water pumped is 10 times higher compared to the quantity required by the SuMeWa|SYSTEM^®^ [45].

### 3.8. Operation of the Treatment System

Due to high hardness levels calcareous deposits formed at the electrolytic cell despite the applied polarity inversion. This caused a reduced cell current especially in P3 with subsequent reduction of ORP and FAC levels and required manual cleaning of the electrolytic cell. Online monitoring allowed identifying these incidents. All sensors and electrodes were still fully functioning after the pilot trail.

The pilot test showed periodical breakthrough of iron from the filter during P1–P3 when a single filter was operated. This went along with increased arsenic effluent concentrations. Backwash was done once per day at a flow rate of 730 L/h for 2 × 7 min and has shown to be sufficient for complete sludge removal. The volume of 170 L of backwash water made up 13% of the total water produced. The arsenic concentration of the water discharged from the sludge tank accounted to 114 ± 57 µg/L, with a one-time value above 200 µg/L. This effluent concentration would cause a daily release of 15.2 mg arsenic per day into the environment, which accounts to 12 µg per liter of treated water. The sludge volume accumulated within the first 212 days during operational phase to 181 L with a water content of 97.4% resulting in a dry matter mass of 4.75 kg or 17.3 mg per liter of water treated.

## 4. Conclusions

The arsenic removal rate of the tested SuMeWa|SYSTEM^®^ setting could be improved along the field test from 80 to 94% by increasing the current density at the electrolytic cell (+8%) between P1 and P2 and by the installation of a second filter in P4 (+6%). These improvements are substantial considering the already low concentrations of arsenic in the effluent prior to those modifications. Total arsenic concentrations of 10 ± 4 µg/L could be achieved, allowing full compliance with the currently given local guideline values of 50 µg/L. The stringent WHO guidelines could be maintained most of the time during the final operation phase P4, when the setting achieved stable removal rates under varying arsenic inlet concentrations. Fe/As ratios during P4 of 34:1 were well below the >40:1 usually required to achieve arsenic concentrations <50 µg/L under field conditions. Considering the high concentration of competing phosphate the here achieved arsenic concentrations are satisfactory. It is assumed that this improvement is caused by the simultaneous oxidation of iron and arsenic with chlorine, subsequent co-precipitation and a very good iron filtration performance during P4.

The performance of the tested system is encouraging considering that the pilots were tested under challenging in-field conditions and did not require the addition of iron salts or chlorine. Safe ammonium and manganese concentrations were only achieved at ORP values between 700 and 900 mV. This was possible only during P2, when the chlorination breakpoint could be reached. The test has also shown that ORP readings can be used to indicate over- and underfeeding of oxidants with regards to chlorine, ammonium, and iron and manganese concentrations. An integrated ORP reading may therefore be used to automatically adapt the oxidant production rate to the required level and by that taking ever changing source water conditions into consideration. The following operation conditions have been identified to be crucial for optimal arsenic removal and stable system operation:

- Sufficiently high current density at the electrolytic cell allowing the availability of FAC even under harsh source water conditions to assure full iron, arsenic and manganese oxidation and ammonium removal, or ammonium concentrations ≤1 mg/L.
- A Fe/As ratio of >30:1 should be available before oxidant application, especially when competing phosphate is present.
- Well-adjusted filtration and backwash setting to prevent filter breakthrough as well as advanced crystallization of iron oxy-hydroxide, which may lead to discharge of arsenic.
- Prevention of pre-oxidation of source water prior to in situ oxidant production, to prevent crystallization and clogging.

The presented system for arsenic removal operates fully autonomously and neither the supply of chemical and electricity nor the exchange of filtration media was required. The energy demand can be met by the installation of a small solar PV system. Next to the good removal efficiencies these factors constitute major benefits compared to existing removal technologies allowing easy compliance by operators. Once set the system runs with nearly no maintenance requirements and the online monitoring system informs about system status to prevent downtime.

In order to reconfirm the results presented here and to evaluate the impact of different source water conditions on the arsenic removal performance the system setting needs to be operated at different sites under different source water conditions. For these tests an increased cell current to assure breakpoint chlorination, the oxidation of organics and the by-product formation potential, as well as a higher production rate will be considered. With an improved sludge tank setting the discharge of arsenic can be further reduced and evaporative and/or solar drying beds allow the reduction of sludge volume, prior to its safe disposal or utilization.

## Figures and Tables

**Table 1:** Comparison of different oxidants applied for arsenic removal (adapted from [33]).

Oxidant	Advantages	Disadvantages
Chlorine	Relatively low cost Primary disinfection capability Secondary disinfectant residual MnO_2_ media regenerant Oxidizes arsenic in less than 1 min	Formation of disinfection by-products possible Special handling and storage requirements
Permanganate	Unreactive with membranes No formation of disinfection by-products MnO_2_ media regenerant Oxidizes arsenic in less than 1 min	Relatively high cost No primary disinfection capability Formation of MnO_2_ particulates Pink water Difficult to handle An additional oxidant may be required for secondary disinfection
Ozone	No chemical storage or handling required Primary disinfection capability No chemical by-products left in water Oxidizes arsenic in less than 1 min in the absence of interfering reductants	Sulfide and TOC interfere with conversion and increase the required contact time and ozone dose for oxidation An additional oxidant may be required for secondary disinfection

**Table 2:** Chemical oxidant stoichiometry [29,44].

Chlorine Species	As(III)	Fe(II)	Mn(II)	NH_4_^+^
Chlorine (mg Cl_2_/mg)	0.95	0.64	1.29	8–10
Monochloramine (mg NH_2_Cl/mg)	0.69	0.46	0.94	-

**Table 3:** Manufacturer and operational data of the GSP Filter.

Parameter	Data Sheet	Applied in Field Test
Uniformity coefficient	1.6	
Porosity	0.45	
Bulk density	1.4 g/cm³	
Media bed depth	76 cm	66 cm (in P4 2 filters)
Drainage gravel bed depth	15 cm	15 cm (in P4 2 filters)
Service flow rate	5–30 m/h	50–60 L/h
Back wash rate (at 13 °C)	≥30 m/h	730 L/h
Bed expansion	40%	32 cm
Automatic backwash intervals		Once per day
Duration and volume of backwash	Until optically clear	2 × 7 min 170 L

**Table 4:** Groundwater quality from test well during test period in West Bengal 2016–2017 and reference values from the literature.

Parameter	Minimum/Maximum	Mean ± SD	*n*	Literature Values **
As_total_ in µg/L	65/330	187 ± 45	50	62 ± 127
Fe_total_ in mg/L	2.0/7.6	5.5 ± 0.8	51	3.7 ± 5.4
Mn_total_ in mg/L	0.4/3.0	1.5 ± 0.4	41	0.6 ± 0.8
PO_4_ in mg/L	0.7/6.1	2.4 ± 1.3	40	2.1 ± 3.6
NH_4_-N in mg/L	0.7/2.9	1.4 ± 0.5	47	2.0 ± 1.0
Hardness mg/L_CaCO3_	285/495	381 ± 54	32	-
Silica as Si in mg/L	16.8/27.3	20.0 ± 3.6	14	21 ± 6
T in °C	22.1/39.9	27.7 ± 3.7	48	-
O_2_ * in mg/L	1.8/4.5	2.7 ± 0.7	53	-
EC in µS/cm	1299/1087	1165 ± 45	53	-
pH	6.23/7.15	6.79 ± 0.21	53	7.0 ± 0.2
ORP in mV	−135/129	−92 ± 44	52	-

* oxygen intrusion through sampling likely, ** Average values taken from [13] for wells (10–90 m) in Bangladesh (BGS-DPHE database).

**Table 5:** Parameters and methods used for analysis with the AL400 photometer.

Parameter	Wavelength	Method	Range
Fe_total_ in mg/L	530	222: 1,10-Phenanthroline	0.02–3
Mn_total_ in mg/L	560	242: PAN	0.01–0.7
PO_4_-P in mg/L	660	323: Phosphomolybdenum blue Ascorbic acid	0.06–2.5
NH_4_-N in mg/L	610	60: Indophenole	0.02–1
Cl^−^ in mg/L	530	90: Silver nitrate/turbidity	0.5–25
Total Hardness in mg/LCaCO_3_	560	200: Metalphthalein	2–50
Free Chlorine	530	100: DPD1	0.01–6
Total Chlorine	530	100: DPD3	0.01–6

**Table 6:** Energy requirements for water treatment (as in P2 and P4).

Consumer	Power		Energy	
Filtration pump	9	W	213	Wh/day
Inline Electrolysis (P2 and P4)	55	W	1320	Wh/day
Control unit and online monitoring	4	W	96	Wh/day
Backwash Pump (20 min/day)	80	W	27	Wh/day
Total demand			1.64	kWh/day

## Supplementary Materials

Supplementary File 1

## Appendix A

**Table A1:** Results from water quality monitoring, mean values and standard deviation, Kalyani treatment system, September 2016–July 2017.

Parameter	Phase 1			Phase 2			Phase 3			Phase 4		
	Ground Water	Treated Water	Removal	Ground Water	Treated Water	Removal	Ground Water	Treated Water	Removal	Ground Water	Treated Water	Removal
Total Arsenic [µg/L]	202 ± 57	36 ± 20	80%	183 ± 43	24 ± 8	88%	195 ± 35	46 ± 26	76%	165 ± 17	10 ± 4	94%
Total Iron [mg/L]	5.9 ± 0.5	0.2 ± 0.2	96%	5.2 ± 1.0	0.2 ± 0.2	96%	5.5 ± 0.4	0.7 ± 0.8	87%	5.5 ± 0.7	0.03 ± 0.03	>99%
Manganese [mg/L]	1.50 ± 0.64	0.30 ± 0.37	83%	1.79 ± 0.24	0.06 ± 0.05	96%	1.46 ± 0.14	0.61 ± 0.53	58%	1.31 ± 0.06	0.60 ± 0.23	55%
Ammonium [mg/L]	1.31 ± 0.63	0.34 ± 0.24	74%	1.14 ± 0.16	0.18 ± 0.12	84%	1.51 ± 0.27	0.67 ± 0.34	56%	1.86 ± 0.28	0.96 ± 0.23	49%
Phosphate [mg/L]	2.4 ± 1.5	0.7 ± 0.3	81%	3.5 ± 0.9	0.7 ± 0.2	72%	1.4 ± 0.3	0.9 ± 0.5	36%	1.6 ± 0.5	0.4 ± 0.1	75%
Silica [mg/L]	20.4 ± 4.2	19.1 ± 2.4	12.3%	19.5 ± 2.5	17.5 ± 0.5	4.7%	--	--	--	--	--	--
Chloride	--	154 ± 32	--	--	154 ± 49	--	--	154 ± 50	--	--	121 ± 31	--
FAC [mg/L]	--	0.10 ± 0.07	--	--	0.37 ± 0.29	--	--	0.14 ± 0.20	--	--	0.16 ± 0.07	--
Total Cl_2_ [mg/L]	--	0.72 ± 0.83	--	--	0.51 ± 0.32	--	--	0.82 ± 0.64	--	--	1.47 ± 0.62	--
EC [µS/cm]	1188 ± 1147	1163 ± 53	--	1147 ± 22	1102 ± 21	--	1132 ± 14	1089 ± 7	--	1180 ± 48	1146 ± 44	--
pH	6.86 ± 0.06	7.00 ± 0.08	--	6.94 ± 0.12	7.02 ± 0.21	--	6.83 ± 0.07	7.15 ± 0.09	--	6.48 ± 0.15	6.67 ± 0.12	--
ORP [mV]	−92 ± 68	473 ± 66	--	−91 ± 36	701 ± 167	--	−93 ± 19	313 ± 116	--	−97 ± 12	399 ± 176	--
Temperature [°C]	24.8 ± 1.5	24.1 ± 1.4	--	27.9 ± 1.0	27.2 ± 0.9	--	25.4 ± 1.1	25.2 ± 1.2	--	32.5 ± 3.3	32.9 ± 3.4	--
