# Supplementary material for: Arsenic Removal from Groundwater by Solar Driven Inline-Electrolytic Induced Co-Precipitation and Filtration—A Long Term Field Test Conducted in West Bengal
Source: Int J Environ Res Public Health. 2017 Oct 2;14(10):1167. doi: 10.3390/ijerph14101167 (PMC5664668; doi:10.3390/ijerph14101167)
Supplement: Supplementary File 1 [file ijerph-14-01167-s001.pdf]

OPTIMIZING ARSENIC REMOVAL DURING IRON REMOVAL: THEORETICAL AND PRACTICAL CONSIDERATIONS

Lytle, D.A.[1] and Sorg, T.J
U.S. Environmental Protection Agency, ORD, NRMRL, WSWRD, TTEB
26 W. Martin Luther King Dr., Cincinnati, OH 45268

and

Snoeyink, V.L.
Department of Civil and Environmental Engineering, University of Illinois
205 N. Mathews, Urbana, IL 61801

ABSTRACT

New health effects research prompted the United States Environmental Protection Agency (USEPA) to reduce the drinking water standard for arsenic from 0.05 to 0.010 mg/L (10 μg/L), and as a result many drinking water systems (particularly smaller ones) throughout the country will no longer be in compliance. A number of technologies are currently available to remove arsenic from water. In waters that contain natural iron, arsenic removal can be achieved during iron removal, but the effectiveness of iron to remove arsenic depends on many variables. The objective of this study was to identify the operational and water quality factors that impact arsenic removal during iron removal. Bench-scale ("batch" and standard jar) tests were used to evaluate the effects of pH, phosphate, other water chemistry variables, and the oxidant used to oxidize Fe(II) on the removal of arsenic. Treatment operation considerations, including sequence of oxidant addition and contact time, were also considered. Results showed that (1) arsenic removal improves with increasing iron concentration and particle surface area; (2) freshly precipitated iron particles had a much greater capacity to remove arsenic than preformed particles that were formed by oxidation of ferrous iron with either oxygen or chlorine; (3) chlorination, or application of a stronger oxidant, may be necessary to improve arsenic removal at many drinking water treatment plants; (4) the point of strong oxidant addition in the treatment train is important; and (5) the pH and other competing water quality variables such as phosphate play significant roles in the amount of arsenic removed.

Keywords: arsenic, iron, water, optimizing removal, oxidant

BACKGROUND

[1]Corresponding author. Mailing address: U.S. Environmental Protection Agency, 26 W. Martin Luther King Dr., Cincinnati, OH 45268. Phone: (513) 569-7432. Fax: (513) 569-7172. Email: lytle.darren@epa.gov

The US EPA recently reduced the drinking water standard for arsenic from 0.05 to 0.010 mg/L (10 µg/L) (USEPA, 2001a, 2002). This reduction was prompted by new health effects research, which concluded that extended human exposure to this element can cause severe illnesses (including various types of cancers) at much lower levels than previously believed. As a result of this reduced standard, arsenic levels in many drinking water systems (particularly smaller ones) throughout the country are no longer in compliance. In response, researchers are developing and evaluating new technologies and optimizing existing ones in order to help these systems meet the new arsenic standard by the compliance date set for January, 2006.

General Arsenic Chemistry. Arsenic, which is found at varying levels in many groundwaters and some surface waters, can have both natural and anthropogenic sources. In natural environments, high levels of arsenic are generally caused by the leaching of arsenic from certain arsenic-containing minerals, such as arsenapyrite and other various arsenic sulfides and sulfosalts (Kim et al., 2002; USEPA, 2003a). In addition to arsenic-bearing host minerals, discharge from various industries (including mining, petroleum refining, and glass and ceramics manufacturing) can cause arsenic pollution. Pesticides, herbicides, and fertilizers are also known sources of arsenic release (Blakely, 1984).

The valence state and species of inorganic arsenic in water depend upon the water's redox state and pH. As a general rule, the trivalent oxidant form (arsenite), As(III), is normally found in groundwater and the pentavalent oxidation form (arsenate), As(V), is found in surface water. However, this rule does not always hold true in groundwater, where both forms have been found together in the same water source (USEPA, 2003b). In general, under anoxic conditions, As(III) is stable, with nonionic arsenous acid (H_3AsO_3) and anionic arsenite ($H_2AsO_3^-$) species dominant below and above pH 9.2, respectively (Figure 1a). As(V) is dominant in oxygenated waters, existing in the anionic forms of $H_2AsO_4^-$, $HAsO_4^{2-}$, or AsO_4^{3-} over the pH range typically encountered in drinking waters (pH 6 to 10) (Figure1b). Generally, As(III) and As(V) acid-base reactions are assumed to occur instantaneously, whereas changes between oxidation states require indeterminate time periods in natural waters (Schecher, 1991). For example, while the conversion of As(III) to As(V) in oxygenated water is predicted, the rate of this transformation may take days or even months, depending on the specific conditions involved. Although organic arsenic species of arsenic

exist, even when highly concentrated they are generally considered to be of less significance than inorganic species (Edwards, 1994a).

Numerous studies (Cheng et al., 1994; Scott et al., 1995a; Hering et al., 1996; Tokunga et al., 1999) have concluded that As(III) is more toxic, soluble, and mobile than As(V); therefore, investigating the oxidation of As(III) has been the subject of many studies (Jain et al., 1999;USEPA, 2001b). Chemical oxidants, including free chlorine, ozone, and potassium permanganate have been found to successfully oxidize As(III) at rates that are much faster than those produced by dissolved oxygen alone (Eary, 1990; Jekel, 1994). Ghurye and Clifford (USEPA, 2001b) reported that chlorine and permanganate rapidly oxidize As(III) to As(V) in the pH range of 6.3 to 8.3. The authors found that dissolved manganese, dissolved iron, sulfide, and total organic carbon (TOC) slowed the rate, but complete oxidation was still accomplished in less than one minute. Ozone rapidly oxidized As(III) in the absence of sulfide and TOC. When either sulfide or TOC was present, the oxidation rate was slowed and quenched, respectively. In addition, chlorine dioxide had only limited success in oxidizing As(III) and monochloramine was ineffective.

Treatment Technologies for Arsenic Removal. Treatment methods for the removal of arsenic from water include coprecipitation processes using iron and aluminum salts, iron removal, anion exchange, lime softening, reverse osmosis, electrodialysis reversal (EDR), nanofiltration, and adsorption media (USEPA, 2000a).

As noted above, many arsenic removal processes are iron-based treatment technologies, such as chemical coagulation with iron salts, natural iron removal from source waters by oxidation and filtration, and iron-based adsorptive media (Gupta & Chen, 1978; Edwards, 1994a; McNeill & Edwards, 1995; Scott et al., 1995b; Holm, 1996; Hering et al., 1996; McNeill & Edwards, 1997). These processes are particularly effective at removing arsenic from aqueous systems because iron surfaces have a strong affinity to adsorb arsenic. As a result, the adsorption and coprecipitation of arsenate and arsenite on iron oxide surfaces have extensively been investigated (Manceau, 1995; Waychunas, 1996; Sun, 1998; Jain, 1999). Table 1 provides a list of studies that examined the adsorption of arsenic onto various iron solids. Studies have shown that the sorption of arsenic is affected by many factors, including pH, water quality, amount and form of iron present, and the existence of competing irons such as phosphate, silicate, and natural organic matter (NOM) (Andreae, 1979; Azcue & Nriagu, 1993; Edwards, 1994b; Al-Juaid et al., 1994; Borho &

Wilderer, 1996). Research has also shown that arsenic is more effectively removed by these methods when present in the arsenate form, although the success of each removal process varies (Edwards, 1994b; Hering, 1996; Leist et al., 2000).

Arsenic Removal by Iron Removal and Iron Precipitation. Due to geochemistry, many arsenic-containing groundwaters also contain significant levels of iron, which is typically in the reduced, i.e., dissolved, Fe(II) state. In these cases, conventional iron removal processes can be used to reduce arsenic by taking advantage of the surface adsorptive capacity of natural iron particles that are produced following the oxidation of Fe(II). The capacity to remove arsenic during iron removal depends largely on the amount of arsenic and natural iron present in the source water. Sorg (2001) proposed an arsenic treatment strategy screening guide (Figure 2), which is based on iron to arsenic concentrations in the source water relative to a 20:1 ratio (USEPA, 2001a; USEPA, 2002). This ratio equates to 1 mg/L iron will remove 50 µg/L arsenic. Section A of Figure 2 represents waters having high iron levels of >0.3 mg/L at an iron to arsenic ratio that is greater than 20:1. Source waters with these qualities are potentially strong candidates for arsenic removal by iron removal. Section B represents waters that have significant levels of iron but it is likely that they do not have enough iron to remove the relatively high levels of arsenic (i.e., iron concentrations >0.3 mg/L with an iron to arsenic ratio that is less than 20:1). "Modified" iron removal, such as adding more iron to the iron removal process can be used to optimize removal under these source water conditions. Section C illustrates source water conditions that have little natural iron (<0.3 mg/L) in which case arsenic removal most likely be approached by other techniques such as adsorptive media, coagulation/filtration, and ion exchange technologies. The treatment strategy screening guide presented in Figure 2 is very basic, and the removal capacities depicted are based on optimum adsorptive processes and operational conditions for As(V). Despite the fact that removal of natural iron can be an effective means of removing arsenic from water, little information is available regarding this subject.

McNeill and Edwards (1995) conducted a survey of arsenic removal in five Fe-Mn removal plants. Two of the plants reported only 0.01 mg Fe/L in the source water (i.e., manganese removal systems). Three of the plants reported between 1.5 to 3.6 mg Fe(II)/L and 20.5, 5.2, and 3.2 µg As/L in the source water. Arsenic removal efficiencies of 8 to 95% were obtained in the plants having greater than 1.5 mg Fe(II)/L in their source water. Significant levels of arsenic removal were not

achieved in the two systems that had very low levels of iron (Mn removal plants). Using adsorptive modeling alone (a conservative approach), Edwards (1994a) predicted that by removing 2 mg Fe/L (initially as Fe(II)) from a water with an initial As(V) concentration of 10 μg/L a 0.75 μg/L soluble effluent arsenic concentration can be achieved. The model also predicts that 1 mg/L of Fe(II) is capable of adsorbing 83% of a 22 μg/L As(V), producing soluble arsenic concentrations of about 3.5 μg/L. In similar work, Clifford and Lin (1991) reported that 60% removal of As(V) was removed by oxidation and coprecipitation of 2 mg Fe(II)/L.

The U.S. EPA evaluated the long-term removal of arsenic from two full-scale iron removal plants, Plant A and Plant B (USEPA, 2000b). Plant A had a treatment capacity of 6.1 million liters per day and a treatment train consisting of aeration, chlorination, sedimentation, filtration, softening, and postchlorination. Total arsenic concentrations at Plant A were reduced from an average of 20.3 μg/L to 3.0 μg/L during the removal of 2.28 mg/L iron.

Plant B treated 5.3 million liters and the treatment train consisted of chlorination, reaction vessels, filtration vessel, and blending. By removing 1.14 mg/L of iron, Plant B decreased the arsenic from an average of 48.5 μg/L to 11.9 μg/L. Differences in arsenic removal levels between the two plants were attributed to the amount of iron in the source waters. Consequently, it was suggested that increasing the amount of iron at Plant B (using a coagulant like ferric chloride) would have improved arsenic removals.

Although natural iron removal processes and iron coagulation/filtration processes are not identical water treatment processes, arsenic removal during each are mechanistically similar in that dissolved Fe(II) oxidation leads to the formation of iron hydroxide solids that coprecipitate or adsorb with arsenic. Bench and full scale studies have investigated these processes of arsenic removal.

The removal of As(V) by iron coagulation/filtration is more effective than As(III) removal (Sorg & Logsdon, 1978; Sorg, 1993; Edwards, 1994b; Hering, 1996). For both forms of arsenic, removal has been shown to be dependent upon iron concentration; removal increased as concentration increased (Gulledge & O'Conner, 1973; Sorg & Logsdon, 1978; Hering, 1996). Arsenic removal of As(III) or As(V) has also been shown to be independent of initial arsenic concentration at levels important in drinking water (Edwards, 1994b; Chen et al., 1994; Hering, 1996). Investigators have reported that As(III) removal during iron coagulation is relatively unaffected by pH (Sorg & Logsdon, 1978; Hering, 1996). As(V) removal during iron coagulation

has been shown to be less effected by pH at or below 8; however, pH levels above 8 result in decreases in removal efficiencies (Sorg & Logsdon, 1978; Edwards, 1994b).

Optimizing Arsenic Removal During Iron Removal. Iron removal in its simplest form involves the oxidation (typically aeration) of soluble iron, Fe(II), to the relatively insoluble Fe(III) form. If manganese is also present, a stronger oxidant, such as potassium permanganate or chlorine, is necessary to oxidize the manganese. Arsenic removal by iron removal processes is achieved through two primary mechanisms: adsorption and coprecipitation (Benefield & J.S., 1990). Adsorption involves the attachment of arsenic to the surface of Fe(III) particles, where coprecipitation involves adsorption and entrapment of arsenic within growing Fe(III) particles by inclusion, occlusion, or adsorption. The capacity to remove arsenic during iron removal is dependent on a number of factors, including water quality, operating conditions, and treatment flow processes. Redox relationships between arsenic, iron, and oxidants are particularly important to consider when optimizing the removal of arsenic during iron removal.

Oxidation of Fe(II) by oxygen (aeration) is well understood and is strongly dependent on the pH of water (Stumm & Lee, 1961). In the proper pH range and with sufficient contact times, Fe(II) oxidation can be achieved by aeration. When followed by filtration, aeration is an effective approach to remove iron from groundwater. Aeration, however, does not effectively oxidize either Mn(II) or As(III). Although some As(III) will absorb onto iron surfaces, the amount is low compared to As(V) (Sorg, 1993; Edwards, 1994b; Hering, 1996). Therefore, the use of a strong oxidant, such as potassium permanganate or free chlorine, will oxidize As(III) to As(V), thereby improving arsenic removal.

Research has shown that pre-formed Fe(III) particles have less capacity to remove As(V) than iron particles formed in the presence of As(V). Edwards (1994b) reported that preformed iron hydroxides only reached a maximum adsorption density of 0.1 M As(V)/M iron hydroxide solid in comparison to a maximum adsorption density of 0.5 to 0.6 for iron hydroxides achieved in the presence of As(V). Edwards attributed such differences to mechanisms that were in place: strictly surface adsorption versus adsorption/coprecipitation.

Hering et al.(1996) examined the water quality factors that affect arsenic removal during iron coagulation and adsorption to preformed hydrous ferric oxides (HFO). Based on experimental results and surface complexation modeling, the author demonstrated that although adsorption is an

important mechanism, it is not the only mechanism controlling arsenic removal during coagulation. Such a case is analogous to the condition in a natural iron removal aeration/filtration plant that applies aeration to oxidize iron, followed by chlorination or permanganate to oxidize As(III), i.e., iron particles formed prior to As(III) oxidation.

The point of strong oxidant addition is, therefore, important with respect to optimizing arsenic removal. Arsenic removal has been observed to increase during this sequence of treatment steps, as opposed to air oxidation followed by chemical oxidation of both Fe(II) and As(III) at the same time. Consequently, oxidation of dissolved Fe(II) and arsenic, As(III), should occur at the same time to maximize arsenic removal.

The need for contact time (contact basin) following oxidant addition is debatable. Strong oxidants oxidize As(III) and Fe(II) very rapidly (AWWARF, 1990;USEPA, 2001b), so time is not critical in the oxidation step process. Based on the experience of the authors, the majority of arsenic is incorporated into the Fe(III) particle during the first several minutes following oxidation. Relatively small amounts of additional arsenic adsorption/removal takes place with extended contact time. Particle development, however, may benefit from extended contact times and may improve filterability. Although there are many systems that operate without contactors, contact time should be examined when anticipated arsenic removals are not achieved. Obviously, water utilities would reduce cost and space requirements by avoiding contactors.

Chemical Oxidation. The stoichiometric amount of oxidant necessary to oxidize As(III), Fe(II), and Mn(II) is important when approximating chemical feed dosage in iron/arsenic removal systems. Avoiding oxidant under-dosing is particularly important in achieving optimal arsenic removal, because under-dosing can result in the incomplete oxidation of As(III), Fe(II), and Mn(II). Table 2 presents the stoichiometric relationships between relevant oxidants and constituents of interest to this research. Given the fact that Fe(II) and Mn(II) typically occur in mg/L concentrations, their oxidant demand dominates relative to arsenic that occurs in μg/L concentrations.

Objective. The objective of this research was to examine the impact of water quality and treatment operation factors on the effectiveness of conventional iron removal process to remove

arsenic from water. The study results provide practical scientific-based guidance on how to optimize arsenic removal efficiency during iron removal process.

MATERIALS AND METHODS

Arsenic bench-scale studies were conducted in a series of controlled single "batch" reactor experiments and standard jar tests. Batch reactor experiments were conducted in a 1.2 liter glass reaction vessel. The top of the cell contained ports for a pH electrode, dissolved oxygen/temperature probe, plexi-glass mechanical stirrer, acid and base feed lines, and a water sampling port. A computer software-controlled dual titrator system (Schott Geräte, Germany) maintained a constant pH by rapidly adding small increments of acid or base to compensate for pH changes caused by the addition of iron and chemical reactions. The computer software (Jensen Systems, Hamburg, Germany) recorded and maintained pH values and titrant volumes in a data file.

Experiments were initiated by adding 1 liter of oxygen saturated double deionized (DDI) water to the reaction cell. The water was mixed using a mechanical stirrer and plexiglass paddle (with a 19.05mm radius blade) at 20 RPM ($G=3.5\ sec^{-1}$). Appropriate amounts of sodium bicarbonate (to provide buffering), chlorine, and arsenic were then added to the water. The titration system was programmed to the desired pH and started after several minutes of mixing. When the pH stabilized, an initial water sample was drawn from the cell for total arsenic analysis. Ferrous sulfate ($FeSO_4$) was then added to give the desired initial iron concentration (typically 1 mg Fe/L [1.79×10^{-5} M] or 5 mg Fe/L [8.95×10^{-5} M]), depending on the experiment goal. Reduced ferrous iron was used to simulate the natural iron, Fe(II), found in source waters. Modifications to chemical addition sequence and chemical forms (e.g., ferrous or ferric iron, arsenite or arsenate) were made to meet experiment objectives. The titration system maintained the pH through changes brought about by iron addition, oxidation and precipitation, and CO_2 transfer at the water:air interface. At the end of the jar test, two 60 ml samples were collected for ICP analysis: a non-filtered sample and a 0.02 µm filtered sample. Water samples were drawn out of the cell with a syringe, approximately 20 minutes after complete oxidation of Fe(II) had taken place (time determined in an independent study).

Jar tests were performed using a six jar (6-1.5 liter plexiglass jars) test apparatus (Phipps and Bird, PB-700 Jar Tester, Richmond, VA) with synthetic or field-site-test water, when tight water quality control (e.g., pH) was not critical (primarily at field sites using natural waters). Global mixing conditions and experimental protocol of jar testing were identified in experiments using a reaction vessel. Synthetic water was prepared by adjusting 7 liters of oxygen saturated DDI with appropriate amounts of sodium bicarbonate. The pH of each sample was adjusted by adding either 0.6 M HCL or 0.5 N NaOH to achieve the pH test goal. Chlorine or potassium permanganate (if needed) were added at this point as well. The water was mixed using a mechanical stirrer at 20 RPM. Arsenic and iron were then added to the jars. The levels, forms, and sequence of iron and arsenic added were defined by the test objectives (e.g., iron first [either ferrous or ferric] for preformed experiments, or arsenic first [arsenate or arsenite] for fresh particle experiments). Initial arsenic and iron levels were measured. After both chemicals were added, samples were collected and tested by the Hach Benchtop spectrophotometer for total iron. The pH of each sample was also determined and adjusted to the appropriate value. At the end of the jar test, two 60 ml samples were collected for ICP analysis: a non-filtered sample and a 0.02 μm filtered sample. Both samples from each jar were collected approximately 20 minutes after complete oxidation of Fe(II) had taken place for pH 7 and approximately 1 hour afterwards for all other pHs.

Water and Chemicals. Unless otherwise specified, all chemicals used in this study were Analytical Reagent (AR) grade. The amount of ultrapure nitric acid, HNO_3 (Ultrex, J.T. Baker Chemical Company, Phillipsburg, NJ), used to preserve samples for metals analysis was 1.5 mL/L per sample. Dilute 0.6 M HCl (Malinckrodt, Inc., Paris, KY) and 0.5 N NaOH (Fischer Scientific, Fairlawn, NJ) were used to adjust the pH, and sodium bicarbonate ($NaHCO_3$) (Fischer Scientific, Fairlawn, NJ) was used to adjust water quality. Sodium hypochlorite (4 to 6% NaOCl, purified grade) (Fischer Scientific, Fairlawn, NJ) was used to oxidize iron (II), which was added as ferrous sulfate ($FeSO_4 \bullet nH_2O$)[2] (Fisher Scientific, Fairlawn, NJ). Ferric iron was added as $Fe_2(SO_4)_3 \bullet nH_2O$ (Ultrex, J.T. Baker Chemical Company, Phillipsburg, NJ), arsenite as $NaAsO_2$ (Malinckrodt, Inc., Paris, KY) and arsenate as $NaHAsO_4 \bullet nH_2O$ (Malinckrodt, Inc., Paris, KY).

---

[2] The purchased material was listed as $FeSO_4 \bullet 7H_2O$ (Fisher Scientific); however, X-ray diffraction (XRD) analysis and titrometric standardization of stock solution indicated that the original chemical dehydrated over time. Analysis showed that $FeSO_4 \bullet 7H_2O$, $FeSO_4 \bullet 4H_2O$ and $FeSO_4 \bullet H_2O$ were present and that the chemical dehydration accounted for about 10% error in the final stock solution concentration.

Analytical Methods. The pH was measured with a Hach Company (Loveland CO) EC40 benchtop pH/ISE meter (Model 50125) and a Hach Company (Loveland CO) combination pH electrode (Model 48600) with temperature corrections. A two-point calibration with pH 7 and 10 standard solutions standardized the instrument daily (Whatman Hillsboro, OR). Dissolved oxygen (DO) was measured with a Hach Company (Loveland, CO) Model DO175 dissolved oxygen meter and a Model 50180 dissolved oxygen probe. Free and total chlorine were measured with a Hach DR/2000 spectrophotometer (Loveland, CO), using the DPD method (Standard Method, 4500-Cl G) (Hach Company 1990). Arsenic was analyzed with a Thermo Jarrel Ash (Franklin, MA) Model SH-4000 graphite furnace atom adsorption spectrophotometer (USEPA Method 7060A). A Thermo Jarrel Ash (Franklin, MA) 61E® purged inductively coupled argon plasma atomic emission spectrophotometer (ICP-AES) was used to analyze total iron (USEPA Method 200.7). Ferrous iron was measured using the 1,10 phenanthroline method (Hach Company, 1990; APHA-AWWA-WEF, 1995). Total iron was also measured by the same method except that a reducing reagent was also included in the reagent powder pillow provided by the Hach Company to convert Fe(III) to Fe(II). Dissolved inorganic carbon (DIC) was analyzed by a coulometric procedure on a UIC Model 5011 CO_2 coulometer (Joliet, IL) with Model 50 acidification module, operated under computer control. Syringe filters (0.02 μm) (Anotop 25®, Whatman, Inc., Clifton NJ) were used to separate colloidal iron-arsenic from soluble. Filtration studies were conducted in advance to ensure that soluble arsenic adsorption onto the filter media during filtration was not an issue.

Other. Glassware (excluding pipettes) used for the preparation of standards and solutions was cleaned using a 5% solution of Contrad 70©. The glassware was thoroughly rinsed with deionized water. Reused glassware was immediately cleaned by soaking in 10% (v/v) concentrated HNO_3 and rinsed with DDI H_2O. Air displacement micropipettes with disposable tips were used for handling and transferring solutions.

RESULTS

The removal of arsenic during iron removal was evaluated using bench-scale tests conducted in the laboratory and at field sites. Unless otherwise noted, experiments were obtained from batch reactor experiments. Fe(II) was oxidized to Fe(III) particles (referred to as “fresh” iron particles),

either in the presence of arsenic or by adding arsenic approximately 5 minutes following iron particle formation (referred to as "pre-formed" particles). The following results identify several operational and water quality factors that impact arsenic removal during iron removal.

pH dependency. A series of tests were conducted in chlorinated and nonchlorinated (saturated dissolved oxygen) synthetic water that initially contained 100 μg/L As (V), 10 mg C/L of dissolved inorganic carbon (DIC) and 1 mg/L of Fe(II) at 24°C. Fe(II) was added following arsenic addition, which resulted in the formation of "fresh" iron particles. The removal of As(V) in both oxygen alone and chlorinated waters was strongly dependent on pH (Figure 3). Arsenic removal decreased in a near linear fashion from approximately 65% to 15% between pH 7 and 10 in the iron particle system that originated from the oxidation of Fe(II) by oxygen. As(V) removal decreased from nearly 100% at pH 7 and 8 to 30% at pH 10 in the iron particle system that originated from the oxidation of Fe(II) by free chlorine.

Freshly precipitated iron particles. Particles formed from the oxidation of Fe(II) by chlorine had a greater capacity (by more than 40%) to remove As(V) than particles formed from oxygen alone at pH values less than 9 (Figure 3). The improved arsenic removal capacity is analogous to the earlier observations of Lytle et al.(2002), who reported on the removal of phosphate (a chemically similar anion) by iron particles formed under identical conditions were greater when chlorine was used to oxidize Fe(II). The findings were attributed to differences in particle properties, particularly density and surface area, which they explained in a particle growth model that considered oxidation kinetics and Fe(II)-Fe(III) particle interactions.

As(V) removal efficiency during coprecipitation with a Fe(III) salt, ferrous sulfate, between the pH range of 7 to 10 was compared to As(V) removal efficiencies achieved when iron particles were initially formed from Fe(II) (Figure 3). Removal efficiencies were nearly identical to the removal of As(V) achieved when chlorine was used to oxidize Fe(II) over the evaluated pH range. Lytle et al., (2004), showed that the iron properties of the particles formed with chlorine, and from hydrolysis of Fe(III) were similar. Their results are consistent with similarities in arsenic removal results brought about by the two different iron particles. The similarities in As(V) removal suggest similarities in iron particle properties (surface area, mineralogy, etc.).

Iron concentration. As(V) removal during iron removal can be improved by increasing iron concentration to increase the number of surface adsorption sites. For example, increasing the initial Fe(II) concentration from 0.1 to 2 mg/L (pH 7, saturated dissolved oxygen alone, 23° C) linearly increased the removal of 100 µg As (V)/L from 20% to over 80% (Figure 4). Improvement in As(V) removal efficiency can be achieved at all pH values by increasing Fe(II) concentration. Figure 5 illustrates that increasing the initial Fe(II) from 1 to 5 mg/L (no chlorine, 100 µg As(V)/L, 23°C) increased arsenic removal by approximately 40% between pH 7 and 10.

Increasing arsenic concentration while holding iron Fe(II) dose constant had a different impact on arsenic removal and the capacity of iron particles to adsorb arsenic. Increasing the initial arsenic concentration from 60 to 240 µg/L while holding the initial iron concentration at 1 mg/L (10 mg C/L, pH 7.6 to 8.2, 23 ˚C) increased the capacity to remove arsenic linearly (r^2= 0.987) from 73 to 195 µg As/mg Fe (92 to 82% arsenic removal) in chlorinated water (Figure 6). Because the capacity of iron to remove arsenic changes with initial arsenic concentration, predicting arsenic removal is site specific and subject to local variability in arsenic water levels.

The impact of As(V) concentration on the removal capacity is illustrated in the following case study at site A. The source water at site A contained 1.3 mg Fe/L and 129 µg As/L (complete average water chemistry shown in Table 3). The treatment process consists of aeration, chlorination, addition of $KMnO_4$, and the addition of a polymer coagulant aid, a contact basin and filtration. The system is able to achieve a final As level of 18 µg/L or an As(V) removal capacity of 80 µg As/mg Fe on average, although the capacity can reach as high as 100 µg As/mg Fe. One approach to meet the new arsenic standard is to add more iron. If one assumes that the capacity of 80 µg As/mg Fe is independent of initial arsenic concentration, roughly only 0.1 mg/L of additional iron should be needed to reduce 18 µg As/L to below the 10 µg/L MCL. However, jar tests showed that it takes at least 1 mg/L of additional iron, Fe(II) or Fe(III), to achieve the arsenic MCL. Therefore, the ability of 1 mg/L of additional iron at lower initial As(V) concentrations was reduced from 100 µg As/mg Fe to 8 µg As/mg Fe, which indicates that low initial As(V) concentrations require a greater amount of iron to optimize As(V) removal.

Other completing water quality variables. Anions such as phosphate and silicate are known to compete with arsenic for adsorption sites on iron hydroxides and subsequently will reduce arsenic removal. Figure 7 shows the impact of phosphate concentration on As(V) removal during

iron removal by oxidation of Fe(II) with oxygen alone and chlorine at pH 8. Arsenic removal efficiency dropped rapidly from approximately 50% to 15% with the addition of 0.5 mg PO_4/L for oxygen-formed Fe(III) particle systems. Orthophosphate concentrations above 0.5 mg had little additional effect. Phosphate also caused a reduction in As(V) removal when iron particles formed from chlorine oxidation. As(V) removal decreased in an exponential manner from approximately 90% to 20% as phosphate concentration was increased from 0.25 to 3 mg PO_4/L (see Figure 7).

Cations can also impact arsenic removal effectiveness during iron removal although their importance is far less than phosphate. Calcium (80 mg/L) improved As(V) removal by nearly 30% at pH's 7, 8, and 9 (in oxygenated system) (Figure 8). These improvements are thought to be associated with changes in the electrophoretic mobility (zeta-potential) as a result of cation addition. Cations tend to decrease the zeta potential and shift the pH of the zero point of charge (ZPC) to a higher pH. This effect is more favorable for arsenic anions to reach adsorption sites, thus increasing effectiveness of arsenic removal.

Oxidant strength & point of application. Test results showed that As(V) removal by iron removal is optimized when oxidized with both chlorine and oxygen as apposed to being treated with only oxygen (Figure 3). In natural systems containing iron, however, arsenic is typically in the reduced As(III) form. A common method to remove iron is air oxidation of the iron followed by filtration. In such systems, As(III) will not be oxidized by oxygen by the time Fe(II) is oxidized completely or partially in the time-frame of the treatment process. Even though some As(III) will be removed by co-precipitation/adsorption with Fe(III) particles, removal efficiency will be substantially lower than As(V) removal. The removal of As(III) and As(V) over a pH range between 7 and 10 (Fe(II) = 1 mg/L As_{init} = 100 μg/L, DIC = 10 mg C/L 23° C) was compared (Figure 9). The removal of As(III) was much less efficient than As(V) removal at pH values below 9 in oxygenated systems. Less than 25% of As(III) removal was achieved at pH 7 and 8 in comparison with approximately 65% and 50% removal of As(V) at the same pH values. There was not a difference at pH 9, and actually, As(III) was more easily removed at pH 10 than As(V), accomplishing 45% and 20% removal respectively. The shift in As removal was likely due to Fe(III) particle and As chemical charge considerations. When chlorine was used as the primary oxidant, As(III) removal was identical to when arsenic was initially added as As(V) (Figures 3 and 9). Consequently, chlorination (or other strong oxidant) in lieu of oxygen can improve arsenic removal at existing iron

removal plants using oxygen for Fe(II) oxidation.

The point where chlorination is applied relative to the point of aeration (and/or Fe[II] oxidation by background oxygen) is extremely important. When the natural ferric iron, Fe(II), is oxidized by oxygen prior to chlorine feed, "preformed" iron particles (that may contain some As[III], Figure 9) are present when As(III) is oxidized to As(V) by chlorine. In this case, arsenic removal is solely controlled by surface adsorption onto the preformed iron particles rather than a coprecipitation/adsorption mechanism. Jar test results performed at site B with natural water containing 75 µg As(III)/L and 1.4 mg Fe/L provide a good illustration (complete water chemistry in Table 3). The tests were conducted to investigate the impact of oxidant dose (chlorine and potassium permanganate) on arsenic removal. The test data show that when only oxygen was present (1.0 mg O_2/L at test start to 4.2 mg O_2/L at test completion), approximately 15 µg/L of arsenic was removed after 20 minutes of contact time. Arsenic removal dramatically improved when chlorine and permanganate concentrations were used to oxidize the ferric iron and As(III) (Figure 10). Data also shows removal increases as oxidant dose was increased from 0.5 to 2 mg/L. Arsenic levels reached a the same low level of nearly 10 µg/L with both oxidants at a dose of 2 mg/L. The test shows that oxidant concentration must be greater than the stoichiometric amount necessary to oxidize major oxidizable species (As, Mn, Fe, etc.) to maximize arsenic removal. However, when a strong oxidant is added after the iron is oxidized by oxygen, little additional arsenic removal is realized. In this work, 4 mg Cl_2/L was added to the jar that initially did not contain chlorine (i.e., iron particles were preformed). Only an additional 20 µg As/L was removed by adding chlorine.

Contact time. Contact time (time for particle and soluble arsenic to interact prior to filtration) is a factor that can potentially influence arsenic removal during iron removal. Contact time can affect adsorption and oxidation kinetics (As and Fe), and filterability of resulting iron particles. Batch experiments were conducted to evaluate the role of contact time on As(V) removal with fresh iron particles formed in the presence of As(V) and preformed iron particles (pH 8, 10 mg C/L DIC, 1 mg Fe/L 100 µg As/L, pH = 25°C). Interestingly, no arsenic removal was observed with preformed iron particles produced by the oxidation of Fe(II) by oxygen (Figure 11). Furthermore, contact time (up to 120 minutes) had no impact on arsenic removal. Preformed iron particles created by the oxidation of Fe(II) by chlorine did have some capacity to remove arsenic from water.

Removal was time dependent, improving rapidly throughout the first 24 minutes of contact to a maximum removal efficiency of approximately 40%. Additional contact time (up to 120 minutes) did not improve arsenic removal. The removal of arsenic with fresh iron particles increased to approximately 60% and 80% when oxygen and chlorine, respectively were used to oxidize Fe(II). Contact time did not improve arsenic removal in either case. The importance of oxidant type and chlorine addition sequence are reemphasized as well.

CONCLUSIONS AND RECOMMENDATIONS

When an arsenic-containing source water contains natural iron, removing the natural iron can reduce the arsenic concentration with the reduction dependent on the iron to arsenic ratio and water quality. This study demonstrated the effects of water quality and operational factors on arsenic removal during iron removal using bench-scale experiments. This study reinforced the following previous findings: (1) arsenic removal decreases during iron removal with increasing pH and phosphate concentration, and (2) arsenic removal capacity was improved by adding iron under all conditions evaluated. The work also introduced the following newer findings: (1) calcium improved the removal of arsenic although the mechanism was not clear, (2) initial increases in arsenic concentration increased arsenic removal capacity (μg As/mg Fe), and (3) the point of iron particle formation relative the point of As(III) oxidation to As(V) was very important.

Source waters containing both natural iron and arsenic can be treated for arsenic reduction by conventional iron removal processes. Recommendations to optimize arsenic removal when designing new iron removal/arsenic removal plants or modifying existing iron removal plants to increase removal are as follows:

• **Strong oxidant addition** - The form of arsenic–As(III) or As(V)–is very important when designing arsenic removal approaches; therefore, arsenic speciation analysis should always be conducted. When the source water arsenic is in the As(III) form, it will almost always be necessary to convert it to As(V) during treatment by the addition of a strong oxidant such as free chlorine or potassium permanganate. Oxygen (aeration) will not complete the conversion of As(III) to As(V) even though it will oxidize iron. Existing aeration systems that have an arsenic problem will likely need to add chlorine before their aerators as location in the treatment chain is critical (see oxidation protocol modification). Naturally, chemical costs and handling issues will need to be considered,

particularly when strong oxidants have not been used. In addition, some ground waters may have significant levels of organic carbon that might limit the use of chlorine addition for concern of creating elevated disinfection by-products.

• **Oxidation protocol modification**- This work clearly supported the fact that preformed iron particles have little capacity for As(V) removal. Fresh iron particles formed from chlorine in the presence of As(V) are less dense and have a greater capacity to adsorb arsenic. Thus, arsenic removal capacity is optimized when (1) As*(III) and Fe(II) are oxidized at the same time* and (2) a strong, chemical oxidant (e.g. free chlorine) is used. In cases where aeration is already in place, the strong oxidant must be added before aeration to achieve optimum results, i.e. fresh iron particles lend to more immediate removal of arsenic than preformed iron particles.

• **pH adjustment**- Decreasing the pH should improve arsenic removal in most cases. However, pH adjustment involves the addition of an acid such a sulfuric acid or carbon dioxide, which raises handling and safety issues, and introduces up front storage costs and long term chemical costs. In addition, post treatment pH readjustment may be necessary to meet corrosion control objectives.

• **Iron addition**- When the amount of natural iron is insufficient to reduce arsenic below the revised MCL, the addition of iron (ferrous or ferric) will improve arsenic removal. Adding iron provides additional surfaces for arsenic to interact with and improved arsenic removal under all conditions evaluated in this study. Like pH adjustment, however, iron addition involves the addition of an acidic chemical and therefore requires safety, handling, and storage considerations. Supplemental natural iron levels will improve arsenic removal during iron removal; however, several considerations must be made. First, the chemicals are acidic and many of the same issue as observed from adjusting pH may exist depending on the buffer capacity of the water. Secondly, increasing the mass of iron will resulting additional solids in the waste stream.

This research has provided a scientific basis for optimizing iron removal processes as well as iron coagulation for the removal of arsenic from water. Many of these points have been effectively employed at a number of full-scale treatment plants and will be the subject of a separate manuscript. Finally, jar testing is a simple approach to evaluating these treatment approaches at the

site based on the extensive experience of the researchers. Jar testing is a common bench-scale test designed to represent full-scale processes and provide treatment performance predictions, chemical dosing levels, operational protocols, and physical-chemical characterization of the aqueous system.

ACKNOWLEDGMENTS

The authors wish to thank Barbara Francis, Jeremy Payne, Victoria Blackschlager and Keith Kelty for water quality and solids analysis, Christy Muhlen from the U.S. Environmental Protection Agency (USEPA) for her assistance in operating the experimental system, and Kathrine McCullough and Dora Wheeler with Miami University for their review of the manuscript.

Any opinions expressed in this paper are those of the author(s) and do not, necessarily, reflect the official positions and policies of the USEPA. Any mention of products or trade names does not constitute recommendation for use by the USEPA.

REFERENCES

Al-Juaid, S.S., Hitchcock, P.B., Johnson, J.A., and Nixon, J.F., 1994. First Structural Evidence for Complexes Containing Arsadiphospholyl Anions. Crystal Structure of the Iron(II) Complex [Fe([eta]5-C5H5) ([eta]5-C2tBu2AsP2)W(CO)5]. *Journal of Organometallic Chemistry,* 480:1-2:45.

Andreae, M.O., 1979. Arsenic Speciation in Seawater and Interstitial Waters: The Influence of Biological-Chemical Interaction on the Chemistry of a Trace Element. *Limnology and Oceanography,* 24:3:440.

APHA-AWWA-WEF, 1995 (19th ed.). *Standard Methods for the Examination of Water and Wastewater.* American Public Health Association, Washington, DC.

AWWARF, 1990. *Alternative Oxidants for the Removal of Soluble Iron and Manganese.* AWWA Research Foundation, Denver, CO.

Azcue, J.M. & Nriagu, J.O., 1993. Arsenic Forms in Mine-polluted Sediments of Moira Lake, Ontario. *Environment International,* 19:4:405.

Benefield, L.D. & J.S., M., 1990. Chemical Precipitation. *Water Quality and Treatment.*

Blakely, N.C., 1984. Behavior of Wastes Co-disposed with Domestic Solid Wastes. *J. Water Pollut. Control Fed.,* 56:1:69.

Borho, M. & Wilderer, P., 1996. Optimized Removal of Arsenate(III) by Adaptation of Oxidation and Precipitation Processes to the Filtration Step. *Water Science and Technology,* 34:9:25.

Chen, R.C., Liang, S., Wang, H.C., and Beuhler, M.D., 1994. Enhanced Coagulation for Arsenic Removal. *J. Am. Water Works,* 86:9:79.

Cheng, R., Liang, Wang, H. & Beuhler, J., 1994. *J. Am. Water Works,* 86:79.

Clifford, D. & Lin, C.C., 1991. *Arsenic III and Arsenic V Removal from Drinking Water in San Ysidro, New Mexico* United States Environmental Protection Agency EPA/600/2-91/011, Cincinnati, Ohio.

Driehaus, W., M. Jekel, and U. Heldebrandt, 1998. Granular Ferrichydroxide a New Adsorbent for the Removal of Arsenic from Natural Water. *Journal Water SRT - Aqua,* 47:1:30.

Eary, L.E., and J.A. Schramke, 1990. In: Chemical Modeling of Aqueous Systems II. *American Chemical Society Symposium Series* Vol. 416, pp. 379-396.

Edwards, M., 1994a. Chemistry of Arsenic Removal During Coagulation and Fe-Mn Oxidation. *J. Am. Water Works,* 86:9:64.

Edwards, M., 1994b. Chemistry of Arsenic Removal During Coagulation and Fe-mn Oxidation. *J. Am. Water Works,* 86:64.

Fendorf, S., M.J. Eick, P. Grossl, and D.L. Sparks, 1997. Arsenate and Chromate Retention Mechanisms on Goethite. 1. Surface Structure. *Environmental Science and Technology,* 31:2:315.

Fuller, C.C., J.A. Davis, and G. Waychunas (1993) . 1993. Surface Chemistry of Ferrihydrite: Part 2. Kinetics of Arsenate Adsorption and Coprecipitation. *Geochimica et Cosmochimica Acta,* 57:2271.

García-Sánchez, A., A. Alastuey, and X. Querol, 1999. Heavy Metal Adsorption by Different Minerals, Application to the Remediation of Polluted Soils. *The Science of the Total Environment,* 242:179.

Grossl, P.R., M. Eick, D.L. Sparks,, S. Goldberg, and C.C. Ainsworth, 1997. Arsenate and Chromate Retention Mechanisms on Goethite. 2. Kinetic Evaluation Using a Pressure-jump Relaxation Technique. *Environmental Science and Technology,* 31:2:321.

Gulledge, J.H. & O'Conner, J.T., 1973. Removal of Arsenic (V) from Water by Adorption on Aluminum and Ferric Hydroxides. *J. Am. Water Works,* 65:8:548.

Gupta, S.K. & Chen, K.Y., 1978. Arsenic Removal by Adsorption. *Journal WPCF,* March:493.

Hach Company, 1990. *DR/2000 Spectrophotometer Instrument Manual.* Loveland, CO.

Hering, J., CHen, P., Wilkie, M., Elimelch, S., and Liang, J., 1996a. *J. Am. Water Works,* 88:155.

Hering, J.G., Chen, P.-Y., Wilkie, J.A., Elimelech, M., and Liang, S., 1996. Arsenic Removal by Ferric Chloride. *J. Am. Water Works,* 88:4:155.

Holm, T.R., 1996. Removal of Arsenic from Water by Iron Removal Processes, *Proc. AWWA Water Quality Technology Conference*, Boston, MA.

Jain, A., K.P. Raven, and R.H. Loeppert, 1999. Arsenite and Arsenate Adsorption on Ferrihydrite: Surface Charge Reduction and Net OH- Release Stoichiometry. *Environmental Science and Technology,* 33:8:1179.

Jain, A., Raven, K.P. & Loeppert, R.H., 1999. Arsenite and Arsenate Adsorption on Ferrihydrite: Surface Charge Reduction and Net OH Release Stoichiometry. *Environmental Science and Technology,* 33:8:1179.

Jekel, M.R., 1994. Removal of Arsenic in Drinking Water Treatment. *Arsenic in the Environment,* Part 1: Cycling and Characterization.

Kim, M.-J., Nriagu, J. & Haack, S., 2002. Arsenic Species and Chemistry in Groundwater of Southeast Michigan. *Environmental Pollution,* In Press, Uncorrected Proof.

Leist, M., Casey, R.J. & Caridi, D., 2000. The Management of Arsenic Wastes: Problems and Prospects. *Journal of Hazardous Materials,* 76:1:125.

Lytle, D.A. & Snoeyink, V.L., 2002. Effect of Ortho- and Polyphosphates on the Properties of Iron Particles and Suspensions. *J. Am. Water Works,* 94:10:87.

Lytle, D.A. & Snoeyink, V.L., 2004. The Effect of Oxidant on the Properties of Fe(III) Particles and Suspensions Formed from the Oxidation of Fe(II). *J. Am. Water Works,* 96:8:112.

Manceau, A., 1995. The Mechanism of Anion Adsorption on Iron Oxides: Evidence for the Bonding of Arsenate Tetrahedral on Free $Fe(O,OH)_6$ Edges. *Geochimica et Cosmochimica Acta,* 59:17:3647.

McNeill, L.S. & Edwards, M., 1995. Soluble Arsenic Removal at Water Treatment Plants. *J. Am. Water Works,* 87:4:105.

McNeill, L.S. & Edwards, M., 1997. Predicting Arsenate Removal During Metal Hydroxide Precipitation. *J. Am. Water Works,* In Press.

Meng, X.S.B., and G.P. Korfiatis, 2000. Effects of Silicate, Sulfate, and Carbonate on Arsenic Removal by Ferric Chloride. *Water Research,* 34:4:1255.

MINEQL+, 1998. Version 4.5, Environmental Research Software, Hallowell, ME.

Namasivayam, C., and S. Senthilkumar, 1998. Removal of Arsenic(V) from Aqueous Solution Using Industrial Solid Waste: Adsorption Rates and Equilibrium Studies. *Industrial & Engineering Chemistry Research,* 37:12:4816.

Paige, C.R., Snodgrass, W.J., Nicholson, R.V., and Scharer, J.M., 1997. An Arsenate Effect on Ferrihydrite Dissolution Kinetics under Acidic Oxic Conditions. *Water Research,* 31:9:2370.

Peng, F.F., and P. Di, 1994. Removal of Arsenic from Aqueous Solution by Adsorbing Colloid Flotation. *Industrial & Engineering Chemistry Research,* 33:4:922.

Savage, K.S., T.N. Tingle, P.A. O'Day, G.A. Waychunas, and D.K. Bird, 2000. Arsenic speciation in pyrite and secondary weathering phases, Mother Lode Gold District, Tuolumne County, California. *Applied Geochemistry,* 15:1219.

Schecher, B., 1991. *A Chemical Equilibrium Program for PCs*, The Procter and Gamble Company.

Scott, K., Green, J., Do, H., and McLean, S., 1995a. *J. Am. Water Works,* 88:155.

Scott, K.N., Green, J.F., Do, H.D., and McLean, S.J., 1995b. Arsenic Removal by Coagulation. *J.*

Am. Water Works, 87:4:114.

Shokes, T.E., and Möller,G., 1999. Removal of Dissolved Heavy Metals from Acid Rock Drainage Using Iron Metal. *Environmental Science and Technology,* 33:2:282.

Smith, K.S., W.H. Ficklin, G.S. Plumoee, and A.L. Meier, 1992b. Metal and Arsenic Partitioning Between Water and Suspended Sediment at Mine-drainage Sites in Diverse Geologic Settings. *Water-rock interaction*, pp. 443-447, 7th International Symposium on Water-rock Interaction, Park City, Utah, v. 1. Rotterdam, A.A. Balkema.

Sorg, T.J., 1993. Removal of Arsenic From Drinking Water by Conventional Treatment Methods. *Proceedings of the 1993 AWWA WQTC.*

Sorg, T.J., 2001. *Opflow.*

Sorg, T.J. & Logsdon, G.S., 1978. Treatment Technology to Meet the Interim Primary Drinking Water Regulations for Inorganics:Part 2. *J. Am. Water Works,* 70:7.

Stumm, W. & Lee, G.F., 1961. Oxygenation of Ferrous Iron. *Ind. Eng. Chem.,* 53:2:143.

Sun, X., and H.E. Doner, 1998. Adsorption and Oxidation of Arsenite on Goethite. *Soil Science Society of America Journal,* 163:4:278.

Swedlund, P.J., and J.G. Webster, 1999. Adsorption and Polymerization of Silicic Acid on Ferrihydrite, and its Effect on Arsenic Adsorption. *Water Research,* 33:16:3413.

Tokunga, S., Yokoyama, S. & Wasay, S.A., 1999. *Water Environ. Res.,* 71:299.

United States Environmental Protection Agency, 2000a. *Arsenic Removal from Drinking Water by Coagulation/Filtration and Lime Softening Plants*. Report EPA/600/R-00/063, Batelle, Columbus OH.

United States Environmental Protection Agency, 2000b. *Arsenic Removal from Drinking Water by Iron Removal Plants* EPA/600/R-00/086, Office of Research and Development, Cincinnati, OH.

United States Environmental Protection Agency, 2001a. *Federal Register: Final Arsenic Rule* 40 CFR Parts 9, 141, and 142.

United States Environmental Protection Agency, 2001b. *Laboratory Study on the Oxidation of Arsenic III to Arsenic V* EPA/600/R-01/021, Office of Research and Development, Washington DC.

United States Environmental Protection Agency, 2002. *Implementation Guidance for the Arsenic Rule--Drinking Water Regulations for Arsenic and Clarifications to Compliance and New Source Contaminants Monitoring* EPA 816K02018.

United States Environmental Protection Agency, 2003a. *Removal of Arsenic from Drinking Water by Adsorptive Media*. Design Manual EPA/600/R-03/019, National Risk Management Research Laboratory, Cincinnati, OH.

United States Environmental Protection Agency, 2003b. *Removal of Arsenic from Drinking Water by Ion Exchange*. Design Manual EPA/600?R-03/080, Office of Research and Development, Cincinnati, OH.

Vagliasindi, F.G.A., M. Henley, N. Schulz, M.M. Benjamin, 1996. Adsorption of Arsenic by Ion Exchange Resins, Activated Alumina, and Iron-oxide Coated Sands, *Proceedings of Water Quality Technoly Conference*, Vol. 2, pp. 1829-1853.

Waychunas, G.A., C.C. Fuller, B.A. Rea, and J.A. Davis, 1996. Wide Angle X-ray Scattering (WAXS) Study of "Two-line" Ferrihydrite Structures: Effect of Arsenate Sorption and Counterion Variation and Comparison with EXAFS Results. *Geochimica et Cosmochimica Acta,* 57:10:2251.

FIGURES & TABLES

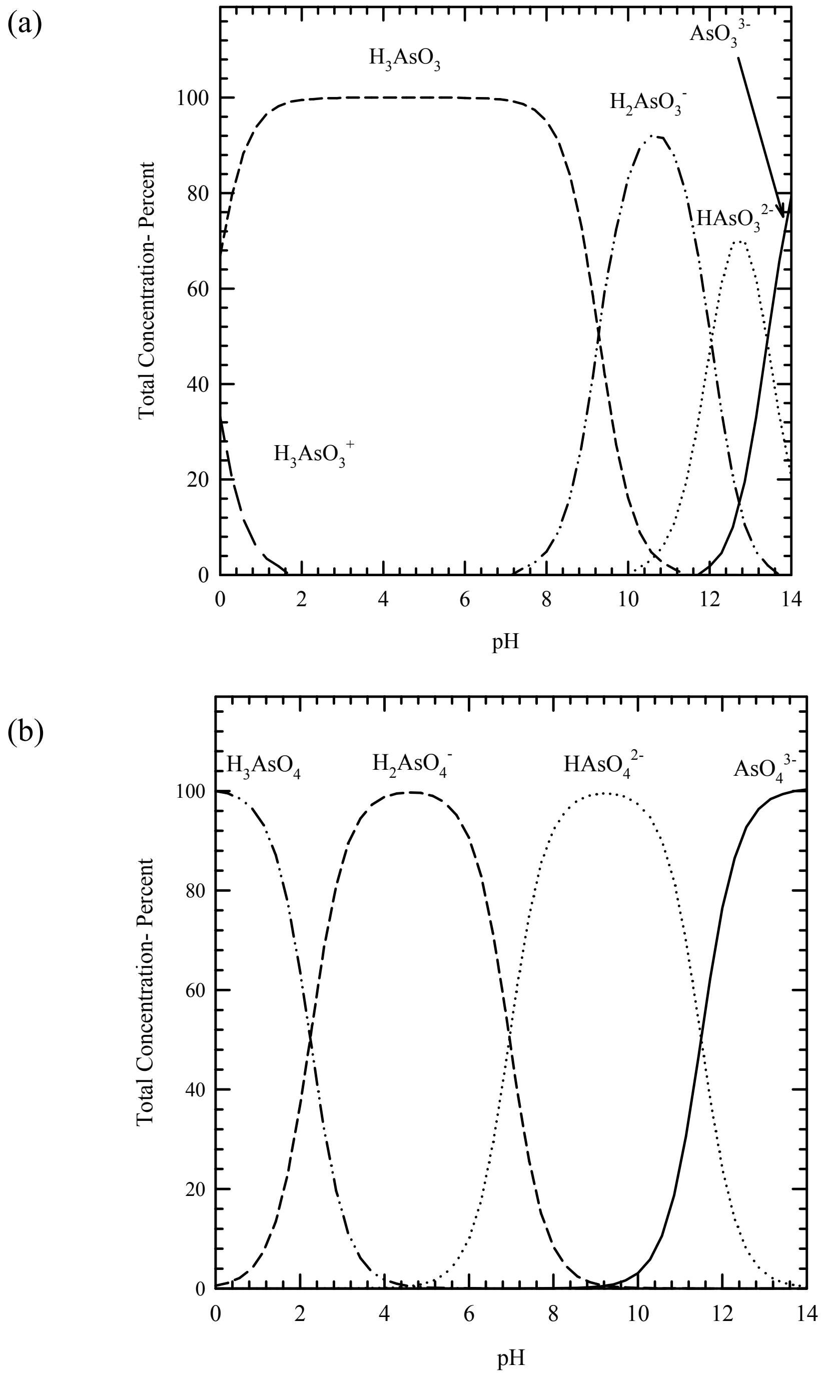

FIGURE 1. The speciation (a) arsenite, and (b) arsenate in water (25ºC). Modeling was performed using MINEQL+ : A chemical equilibrium modeling system version 4.0. (MINEQL+, Version 4.5, Environmental Research Software, Hallowell, ME, 1998).

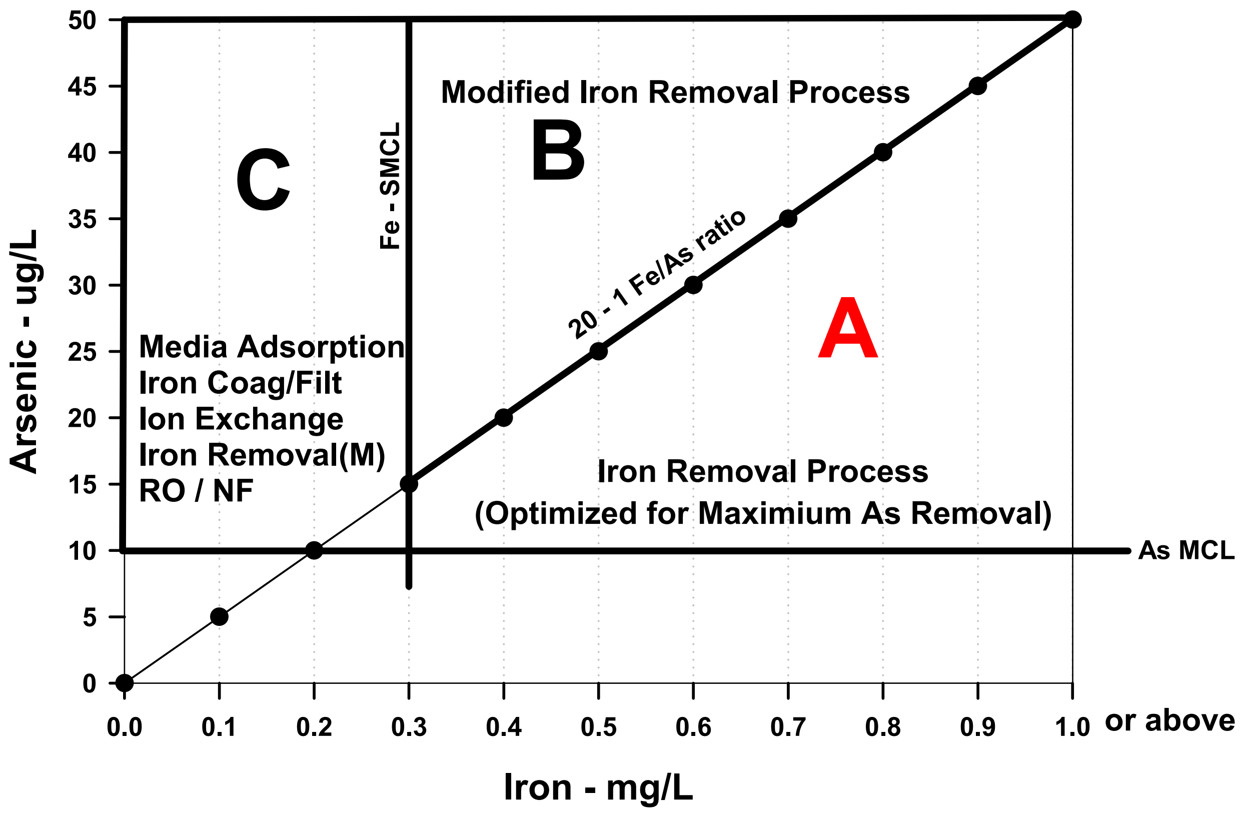

FIGURE 2. Arsenic treatment strategy selection guide as a function of initial arsenic and iron content of water. Based on optimized arsenic removal of As (V).(*Upflow*, 2001)

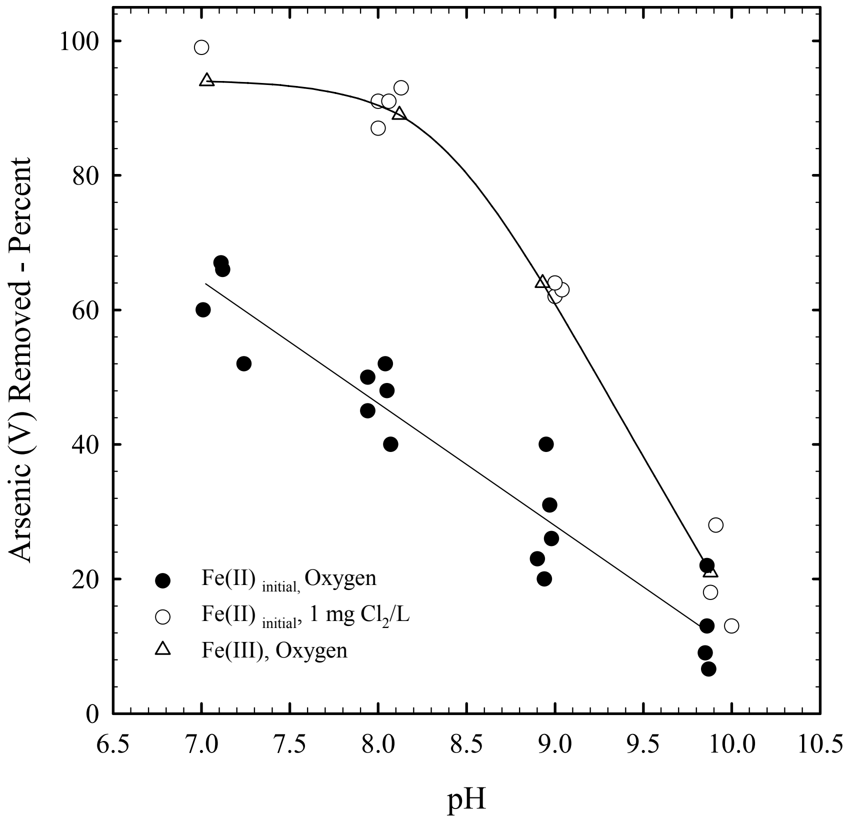

FIGURE 3. The effect of pH, iron and chlorine on arsenic (V) removal with iron (II) and (III) (1 mg Fe/L, 100 µg As(V)/L, 10 mg C/L DIC, O_2= saturated, 24 ^{O}C).

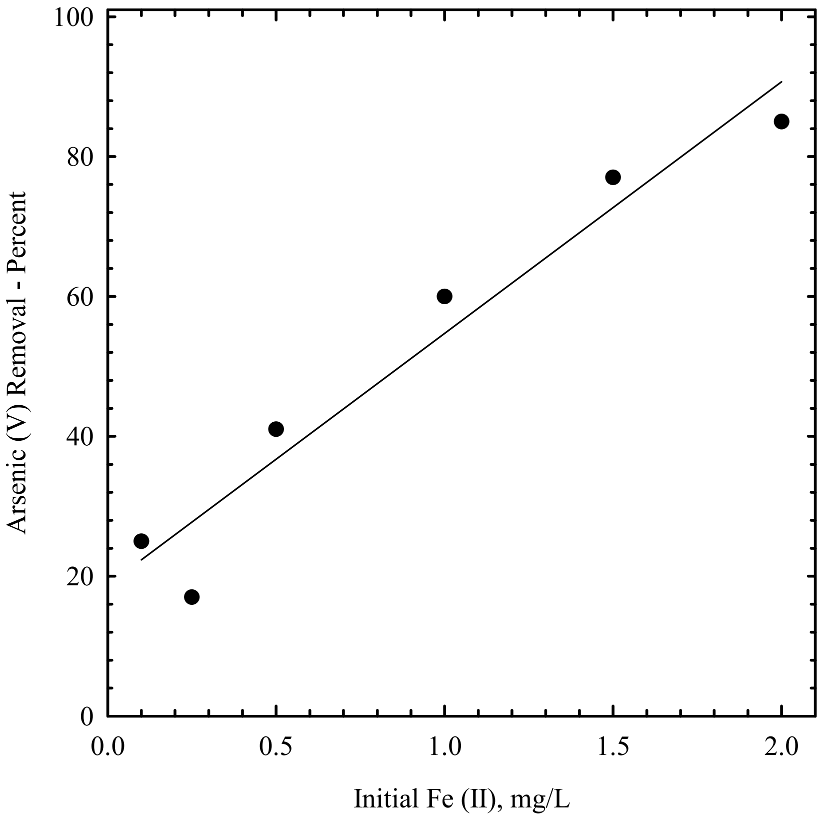

FIGURE 4. The effect of iron (II) dose on arsenic (V) removal (As (V) $_{initial}$ = 100μg/L, 10 mg C/L DIC, pH =7.33 - 7.50, O_2 = saturated, 23° C).

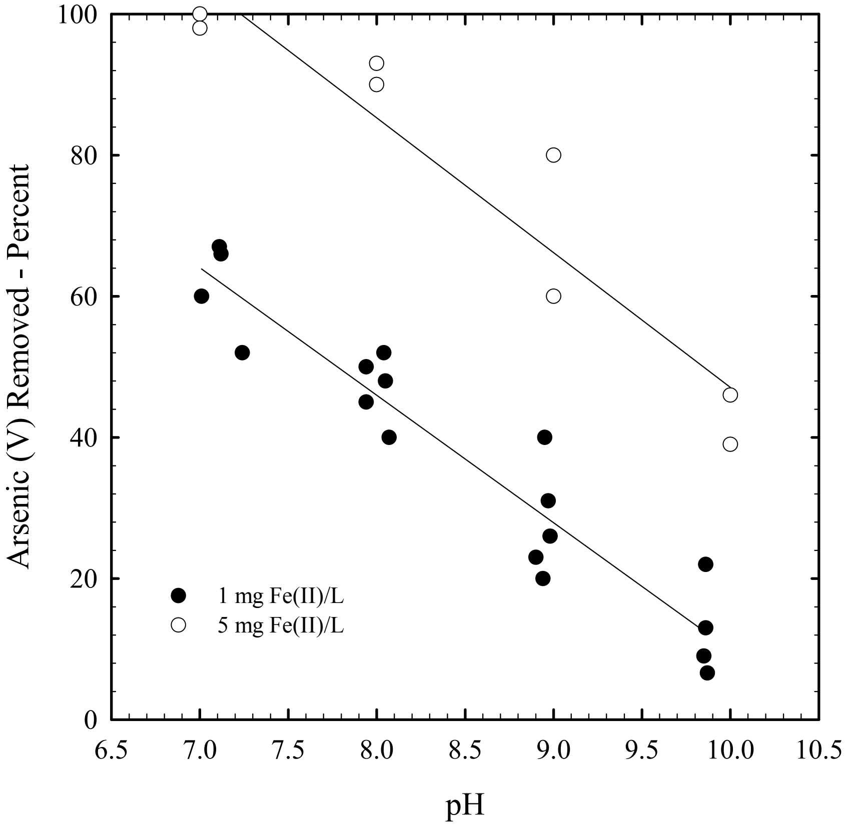

FIGURE 5. The effect of pH and iron (II) dose on arsenic (V) removal (100 μg As(V)/L, 10 mg C/L DIC, O_2= saturated, 24 ^{O}C) .

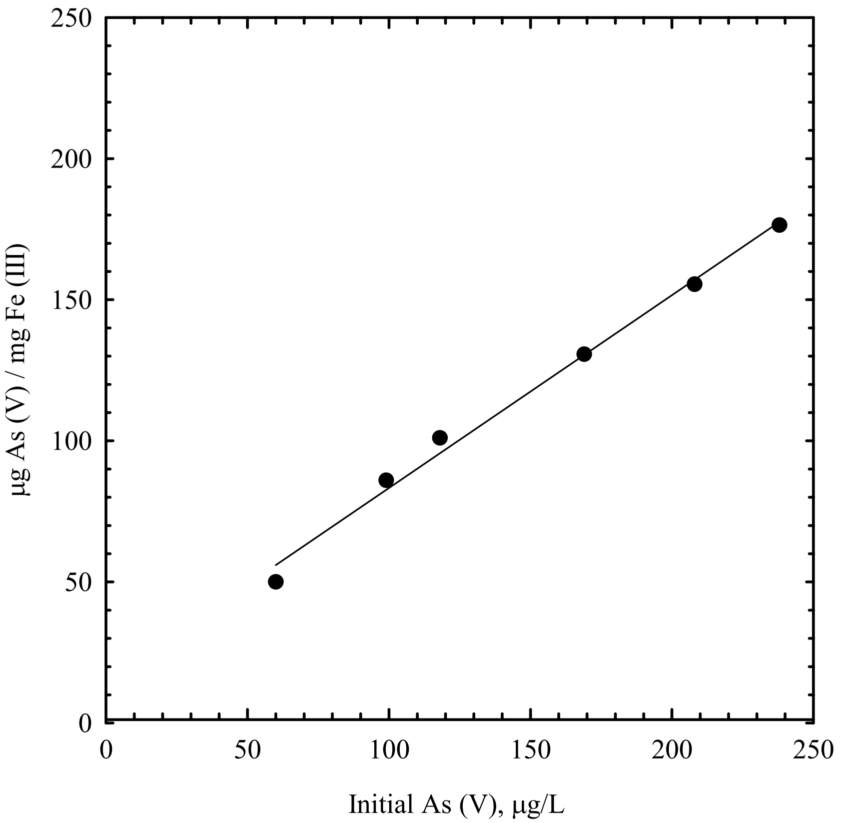

FIGURE 6. The effect of chlorine and arsenic (V) concentration on the capacity of iron to adsorb arsenic (V). (1 mg Fe(II)$_{initial}$/L, 1 mg Cl_2/L, 10 mg C/L DIC, pH= 7.55, 24°C).

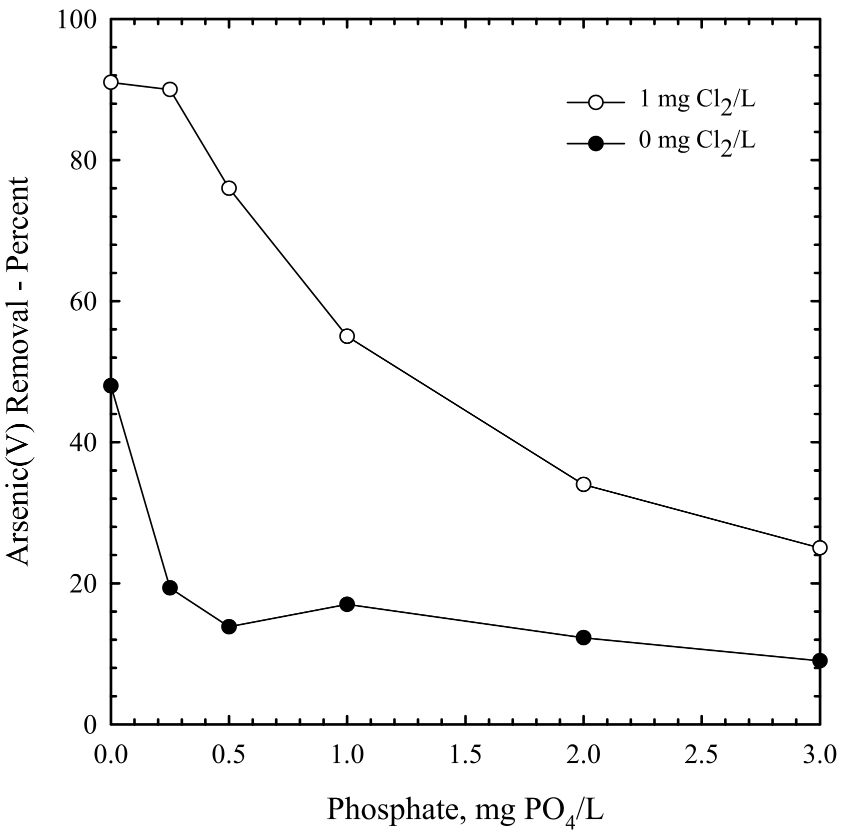

FIGURE 7. The effect of chlorine and phosphate on arsenic (V) removal with iron (III) (10 mg C/L DIC, 1 mg $Fe(II)_{initial}$/L, pH = 8, 100 µg As(V)/L).

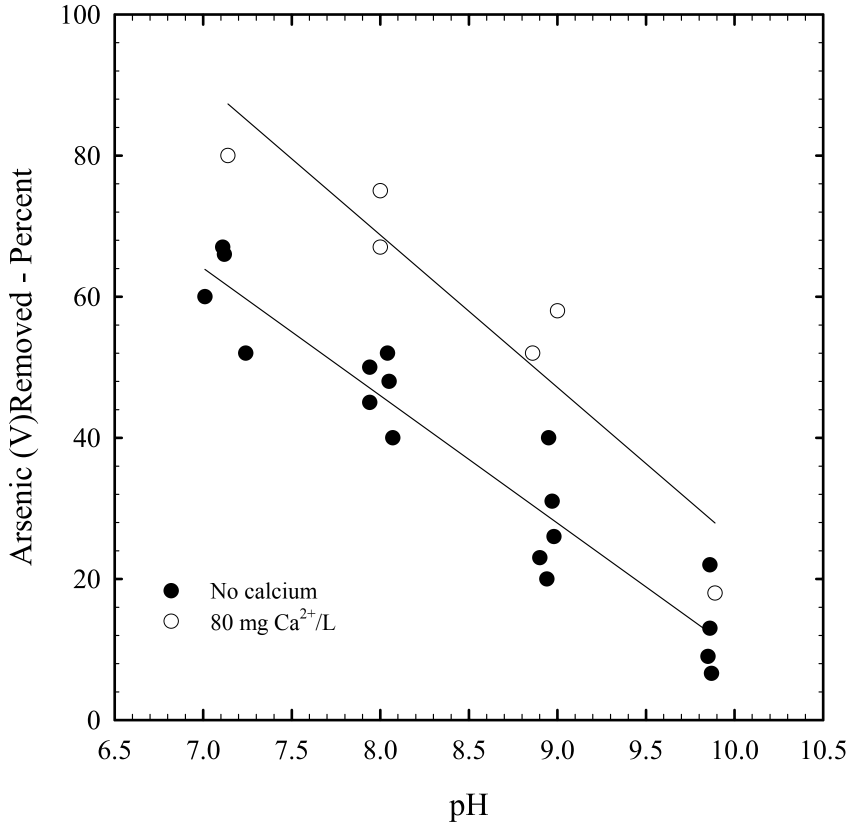

FIGURE 8. The effect of pH and calcium on arsenic (V) removal with iron (III) (1 mg $Fe(II)_{initial}$/L, 100 µg As(V)/L, 10 mg C/L DIC, O_2= saturated, 24 ^{O}C).

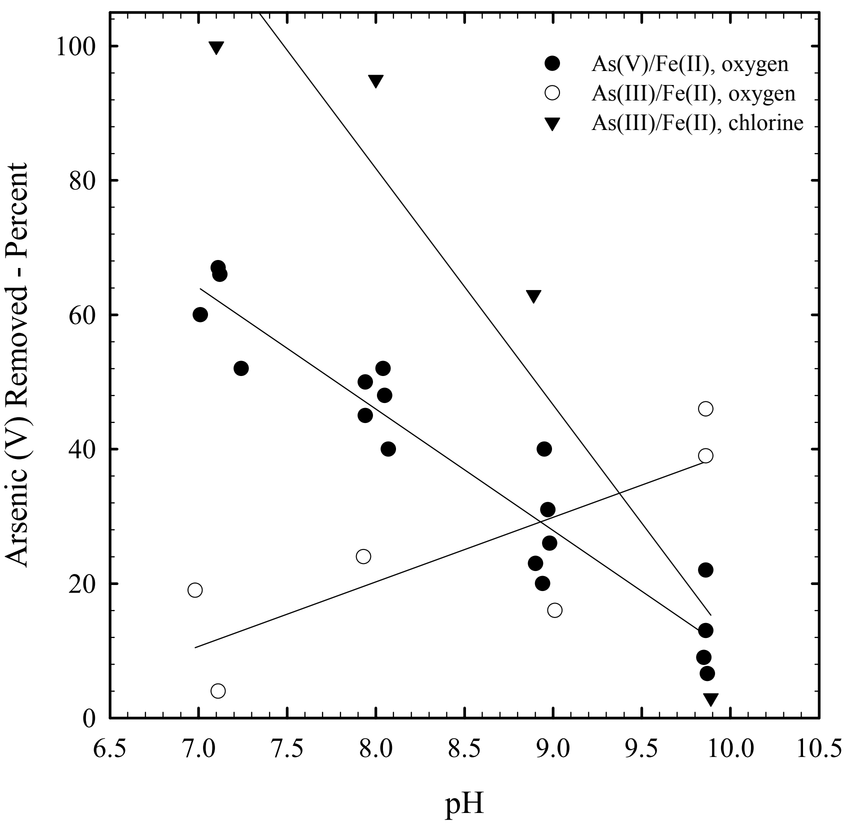

FIGURE 9. The effect of the oxidant, chlorine and oxygen, on arsenic (III) and (V) removal with iron (III) (1 mg $Fe(II)_{initial}$/L, 100 μg As/L, 10 mg C/L DIC, O_2= saturated, 24 ^{O}C).

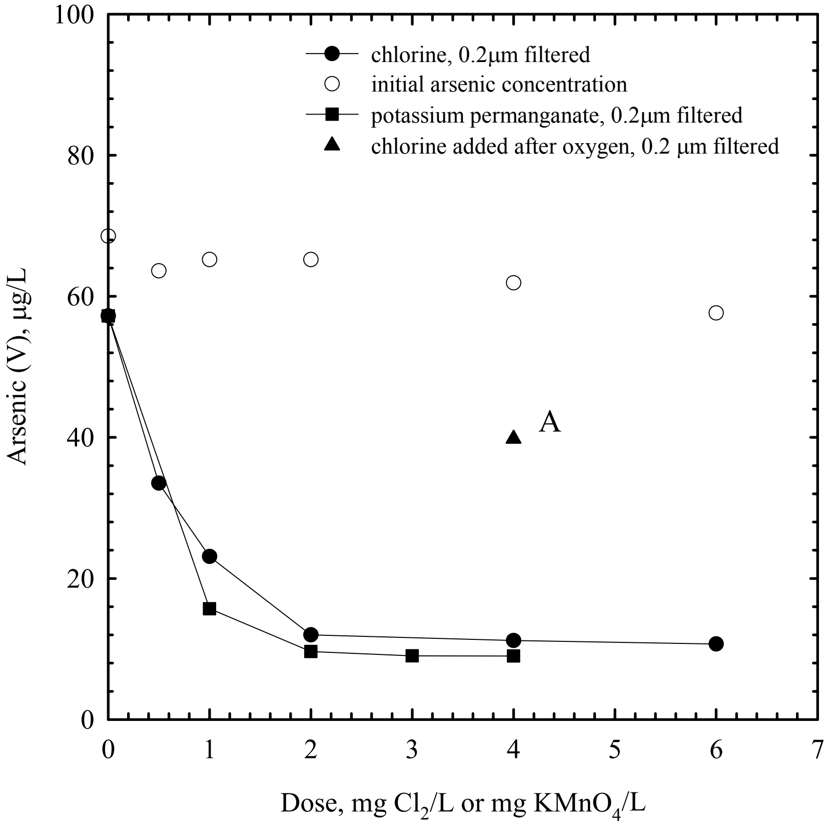

FIGURE 10. The effect of oxidant type and concentration on the removal of arsenic (V) with iron (II) (60-64 µg $As(III)_{initial}$/L, pH = 7.9).

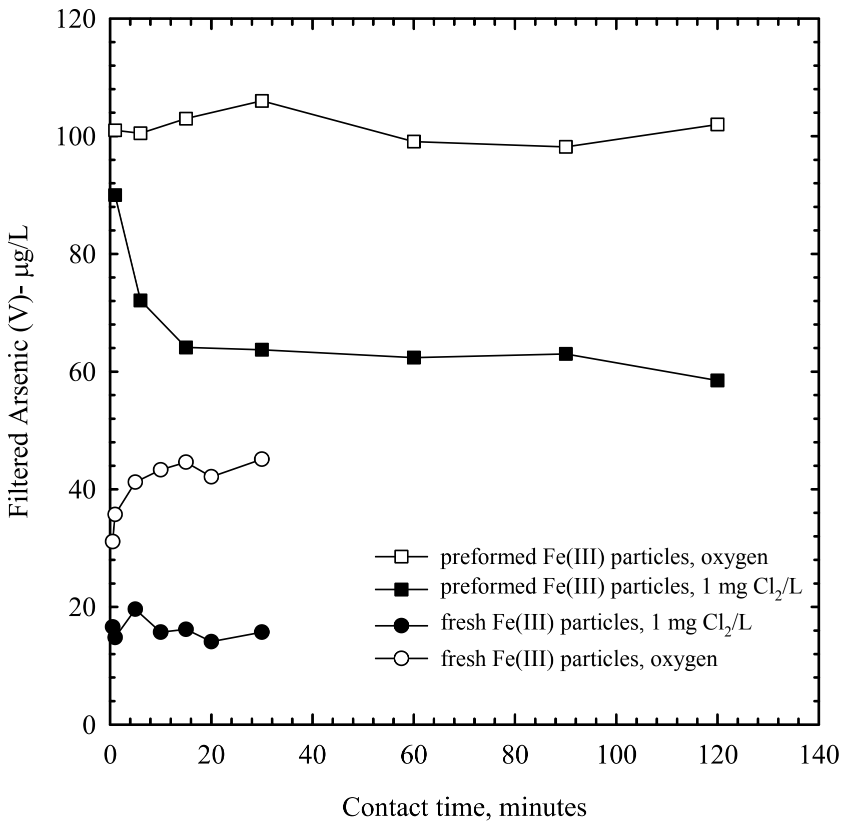

FIGURE 11. The effect of contact time and iron (III) particle formation conditions on arsenic (V) removal with iron (1 mg $Fe(II)_{initial}$/L , 100 µg $As(V)_{initial}$/L , 10 mg C/L DIC, pH= 8, 24°C).

TABLE 1. Adsorption of Arsenic onto Iron-Based Solids.

Arsenic species	Sorbent	Source of Sorbent	Reference
As	Goethite,(α-FeOOH)	Mother Lode District, Tuolumne County, CA	Savage *et al*. (2000)
As(V)	Goethite, (α-FeOOH)	Cerro del Hierro, Sevilla, Spain	García-Sánchez *et al*. (1999)
As(V)	Goethite, (α-FeOOH)	Sierra de la Culebra, Zamora, Spain	García-Sánchez *et al*. (1999)
As(III) and As(V)	Goethite, (α-FeOOH)	Synthetic	Sun and Doner (1998)
As(V)	Goethite, (α-FeOOH)	Synthetic	Fendorf *et al*. (1997)
As(V)	Goethite, (α-FeOOH)	Synthetic	Grossl *et al*. (1997)
As(V)	Granular ferric hydroxide (β-FeOOH)	Synthetic	Driehaus *et al*. (1998)
As	Jarosite, ($KFe_3^{3+}(SO_4)_2(OH)_6$)	Mother Lode District, Tuolumne County, CA	Savage et al. (2000)
As(III) and As(V)	Ferrihydrite ($Fe_2O_3 \cdot 2H_2O$)	--	Swedlund and Webster (1999)
As(III) and As(V)	Ferrihydrite ($Fe_2O_3 \cdot 2H_2O$)	--	Jain *et al*. (1999)
As(V)	Limonite, ($Fe_2O_3 \cdot nH_2O$)	Rio Tinto, Huelva, Spain	García-Sánchez *et al*. (1999)
As	Corroding iron and sulfate reducing bacteria (SRB)	Acid rock drainage	Shokes and Möller (1999)
As	Ferrihydrite,($Fe_2O_3 \cdot 2H_2O$)	Synthetic	Paige et al. (1997)
As(V)	Iron-oxide coated sand	Synthetic	Vagliasindi *et al*. (1996)
As(III) and As(V)	Hydrous ferric oxide (coagulant $FeCl_3$)	--	Hering *et al*. (1996)
As(III)	$Fe(OH)_3$	--	Edwards (1994)
As(V)	Ferrihydrite, ($Fe_2O_3 \cdot 2H_2O$)	Synthetic	Fuller *et al*. (1993)
As	Hydrous ferric oxides	--	Smith *et al*. (1992b)
As(V) and As(III)	$Fe^{III}(OH)_3$	Solution of $FeCl_3$ and HCl (Fisher Scientific)	Meng et al. (2000)
As(V) and As(III)	$Fe^{III}(OH)_3$	Solution of $FeCl_3$ and HCl (Fisher Scientific)	Meng et al. (2000)
As(V)	Fe(III)/Cr(III) hydroxide	--	Namasivayam and Senthilkumar (1998)
As	Ferric hydroxide and Sodium dodecyl sulfate	--	Peng and Di (1994)

TABLE 2. Chemical Oxidant Stoichiometry

	**As(III)**	**Fe(II)**	**Mn(II)**
**Chlorine**	0.95 mg Cl_2/ug As(III)	0.64 mg Cl_2/mg Fe(II)	1.29 mg Cl_2/mg Mn(II)
**Permanganate**	1.06 mg MnO_4^-/ug As(III)	0.71 mg MnO_4^-/mg Fe(II)	1.44 mg MnO_4^-/mg Mn(II)
**Ozone**	0.64 mg O_3/ug As(III)	0.43 mg O_3/mg Fe(II)	0.88 mg O_3/mg Mn(II)
**Chlorine Dioxide**	1.80 mg ClO_2/ug As(III)<br>1-electron transfer<br>0.36 mg ClO_2/ug As(III)<br>5-electron transfer	0.24 mg ClO_2/mg Fe(II)<br>5-electron transfer	2.45 mg ClO_2/mg Mn(II)<br>1-electron transfer
**Monochloramine**	0.69 mg NH_2Cl/ug As(III)	0.46 mg NH_2Cl /mg Fe(II)	0.94 mg NH_2Cl /mg Mn(II)

* Table created from calculations presented in: Ghurye, G. and D. Clifford. United States Environmental Protection Agency, 2001b. Laboratory Study on the Oxidation of Arsenic III to Arsenic V EPA/600/R-01/021, Office of Research and Development, Washington DC.

TABLE 3. Water Chemistry Data for Arsenic Sites

SITE	Location	Sample Location Description	As ug/L	Ca mg/L	Cl mg/L	Fe mg/L	Mg mg/L	Mn mg/L	Na mg/L	Total PO_4 mg/L	SiO_2 mg/L	SO_4 mg/L	Total Alkalinity mg/L $CaCO_3$	pH
A	North Dakota	Raw	129	133	na	1.39	32	0.64	159	na	31	376	na	7.88
		Finished Water	18	132	2.2	0.03	31	0.03	159	na	30	374	na	na
B	Ohio	Raw	69	115	nd	1.38	59	0.163	11	na	na	10	210	7.9
		Finished Water	8	48	na	0.01	14	0.363	10	na	11	na	na	na
		Distribution	5	111	na	0.07	57	0.292	11	na	na	1	213	7.7

na = Not Available
nd = Non Detect
